# Supplementary material for: Shallow-level defect passivation by 6H perovskite polytype for highly efficient and stable perovskite solar cells
Source: Nat Commun. 2024 Jul 4;15:5632. doi: 10.1038/s41467-024-50016-6 (PMC11224362; doi:10.1038/s41467-024-50016-6)
Supplement: Supplementary file 1 — Supplementary Information [file 41467_2024_50016_MOESM1_ESM.pdf]

Supplementary information for

Shallow-level Defect Passivation by 6H Perovskite Polytype for Highly Efficient and Stable Perovskite Solar Cells

Hobeom Kim[1,2,10*], So-Min Yoo[1,3,10], Bin Ding[1,10], Hiroyuki Kanda[1], Naoyuki Shibayama[4], Maria A. Syzgantseva[5,6], Farzaneh Fadaei Tirani[1], Pascal Schouwink[1], Hyung Joong Yun[7], Byoungchul Son[8], Yong Ding[1], Beom-Soo Kim[3], Young Yun Kim[3], Junmo Park[2], Olga A. Syzgantseva[5], Nam Joong Jeon[3*], Paul J. Dyson[1*], Mohammad K. Nazeeruddin[1,9*]

[1]Institute of Chemical Sciences and Engineering, École Polytechnique Fédérale de Lausanne (EPFL); Lausanne, CH-1015, Switzerland.

[2]School of Materials Sciences and Engineering, Gwangju Institute of Science and Technology (GIST); Gwangju, 61005, Republic of Korea.

[3]Division of Advanced Materials, Korea Research Institute of Chemical Technology (KRICT); Daejeon 34114, Republic of Korea.

[4]Faculty of Biomedical Engineering, Graduate School of Engineering, Toin University of Yokohama; Yokohama, 225-8503, Japan.

[5]Department of Chemistry, Lomonosov Moscow State University; Moscow 119991, Russia.

[6]Department of Physics, Mendeleev University of Chemical Technology, Moscow, 125047 Russia.

[7]Research Center for Materials Analysis, Korea Basic Science Institute (KBSI); Daejeon, 34133, Republic of Korea.

[8]Center for Research Equipment, Korea Basic Science Institute (KBSI); Daejeon, 34133, Republic of Korea.

[9]Center of Excellence for Advanced Materials Research (CEAMR), King Abdulaziz University; Jeddah, 21589, Saudi Arabia.

[10]These authors contributed equally.

Email: hobkim@gist.ac.kr, njjeon@krict.re.kr, paul.dyson@epfl.ch, mdkhaja.nazeeruddin@epfl.ch

Supplementary Note 1

The incorporation of excess PbI_2 and/or MACl into perovskites has been exploited to achieve high quality polycrystalline perovskite films. In general, the amount of excess PbI_2 does not exceed 10% and the amount of MACl is around 40 mol%[1–12]. In contrast, we used a higher concentration of PbI_2 and MACl in order to form the 6H polytype perovskite. The amount of excess PbI_2 and MACl used in the $FAPbI_3$–based perovskites in previous works and our work is summarized in Supplementary Tables 1 and 2.

Supplementary Table 1.

Composition of perovskite precursors with excess PbI_2 for $FAPbI_3$–based perovskite and the ratio of PbI_2 and FAI.

Ref.	[1]	[2]	[3]	[5]	[6]	[7]	**Our work**
**PbI_2**	1.1475 M	0.9434 M	1.11 M	1.47 M	1.3585 M	1.54 M	1.44 M
**FAI**	1.093 M	0.8925 M	1.05 M	1.4 M	1.235 M	1.4 M	1.2 M
**Excess PbI_2 (%)**	5%	5.7%	5.7%	5%	10%	10%	20%

Supplementary Table 2.

Concentration of MACl in perovskite precursors used to prepare $FAPbI_3$–based perovskite.

Ref.	[6]	[7]	[8]	[9]	[10]	[11]	[12]	**Our work**
**MACl**	29 mol%	35 mol%	32 mol%	35 mol%	35 mol%	40 mol%	36.7 mol%	55 mol%

In studies using excess PbI_2, the improvement in the morphology (e.g. increase in grain size), and the crystallinity of perovskites is used to explain the suppression of non-radiative recombination[1–5,13]. In contrast, here excess PbI_2 is used to induce the formation of the 6H polytype perovskite that affords hetero-polytypic perovskite.

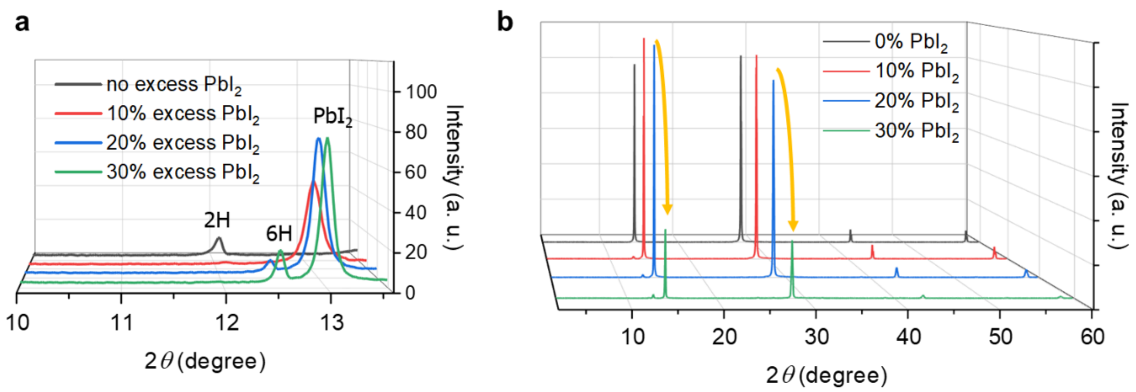

Supplementary Fig. 1.

X-ray diffraction (XRD) patterns of the perovskite films as a function of the concentration of PbI_2 with a fixed concentration of MACl of 55 mol%. **a**, Magnified XRD patterns of the films in the low 2θ angle region. **b**, The overall XRD patterns of the perovskite films.

To show the conditional formation of the 6H polytype, XRD analysis was performed on perovskite films with different concentrations of excess PbI_2 from 0% to 30% in $FAPbI_3$ with a fixed concentration of MACl of 55 mol%. Without excess PbI_2, a diffraction peak at 12.1° is observed, which may be attributed to the formation of non-perovskite 2H phase. The addition of 10% excess PbI_2 suppresses the formation of the 2H phase but did not result in the formation of the desired 6H phase. It was only with the addition of more than 20% excess PbI_2 that the 6H phase is observed. However, the addition of 30% excess PbI_2 significantly reduces the diffraction intensity of the cubic polytype (3C) $FAPbI_3$ at 14.3 and 28.4°. Therefore, to realize highly crystalline 3C/6H hetero-polytypic perovskites, while maintaining high crystallinity of 3C phase, it is necessary to use 20% of excess PbI_2.

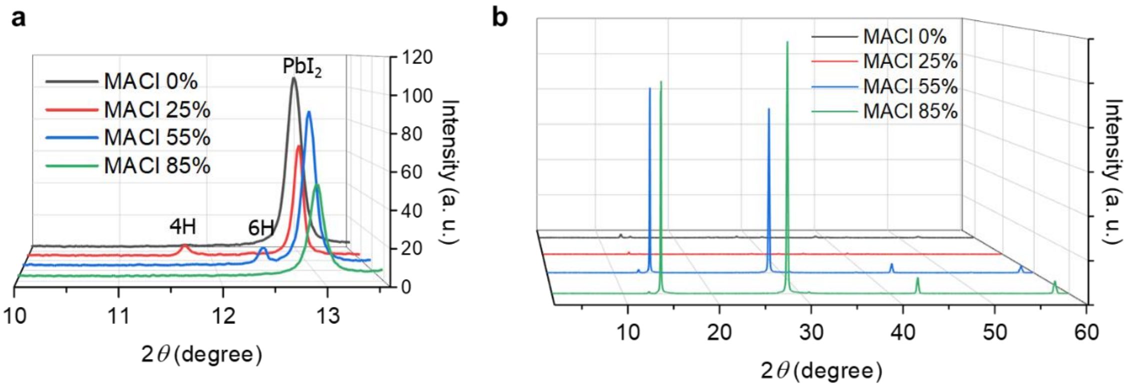

Supplementary Fig. 2.

X-ray diffraction (XRD) patterns of the perovskite films as a function of the concentration of MACl with a fixed concentration of PbI_2 (20% excess). **a**, Magnified XRD patterns of the films in the low 2θ angle region. **b**, The overall XRD patterns of the perovskite films.

The formation of the 6H polytype also depends on the concentration of MACl. Thus, the impact of various MACl concentrations on perovskite films was evaluated. XRD patterns of perovskite films with MACl concentrations ranging from 0 to 85% are shown in Supplementary Fig. 2. In the absence of MACl, a diffraction peak corresponding to unreacted PbI_2 is observed at 13.0°. The addition of 25 mol% of MACl led to the appearance of a new diffraction peak at 11.7°, which may be associated with the formation of the 4H phase, composed of both face- and corner-sharing PbI_2 with a greater proportion of face-sharing in contrast to the 6H phase. It is worth noting that the intensity of the 3C phase with the addition of < 55 mol% MACl is so weak that the compositions are not suitable for use as a light absorber. Conversely, the addition of 55 or 85 mol% MACl greatly increases the diffraction intensity of the 3C phase. However, the highest concentration of MACl did not result in the formation of 6H phase, with the optimal amounts being 55 mol% MACl with 20 mol% excess PbI_2 to attain highly crystalline 3C/6H hetero-polytypic perovskites.

Supplementary Note 2

As discussed in Supplementary Note 1, the formation of the 3C/6H hetero-polytypic perovskite requires a specific composition including excess PbI_2 along with the high concentration of MACl. The use of excess PbI_2 increases the degree of supersaturation of the precursor solution and intermediate phase, which in turn increases the nucleation rate of perovskites according to von Weimarn theory[14,15]. In general, PbI_2 which consists of edge-sharing $[PbI_6]^{4-}$ octahedra in the intermediate phase of perovskite, is expected to reorganize into a corner-sharing perovskite structure with the intercalation of FA^+ and I^-[16,17]. However, in the presence of excess PbI_2 the degree of supersaturation of the intermediate phase increases, and rapid nucleation of perovskite crystal occurs, leaving insufficient time for edge-sharing PbI_2 to transform into the corner-sharing component. Instead, the resulting relative deficiency of FAI increases the probability of forming face-sharing octahedra, leading to the co-existence of corner-sharing and face-sharing, which ultimately results in the formation of the 6H phase. (Supplementary Fig. 3) This hypothesis is corroborated by a computational study where the removal of FAI from FAI-intercalated PbI_2 rendered Pb-I octahedral face-sharing from edge-sharing[18].

Furthermore, MACl plays a crucial role in the dynamics of the formation of 6H polytype. It has been previously reported that MACl stabilises the $FAPbI_3$ lattice structure[11]. In a stoichiometric $FAPbI_3$ composition without excess PbI_2, Cl^- temporarily occupies I^- sites, thereby stabilising the $FAPbI_3$ in its intermediate phase. However, the Cl^- is subsequently de-doped by I^- during thermal annealing, leading to Cl-free $FAPbI_3$. Concurrently, the incorporation of MA^+ into $FAPbI_3$ induces lattice contraction, attributed to its higher dipole moment and smaller size compared to FA^+. However, the formation of the 6H phase was not observed, indicating that the resulting lattice distortion was within tolerable limits[11,19,20].

In contrast, our study confirms the formation of the 6H polytype. This distinction arises from the incorporation of both MA^+ and Cl^- in $FAPbI_3$. Such dual incorporation might further promote lattice contraction, reduce Pb–Pb distances between the octahedra, and facilitate the formation of face-sharing octahedra. Ultimately these changes might drive the phase transformation from the 3C to the 6H polytype.

To gain further insights, X-ray photoemission spectroscopy (XPS) of the perovskite films was performed with varying levels of excess PbI_2 from 0 to 30%, while maintaining the concentration of MACl at 55% (Supplementary Fig. 4). With 0 or 10% excess PbI_2, no Cl signal was observed.

In contrast, when the excess PbI_2 reaches 20%, the films start to exhibit distinct Cl signals, suggesting that there is a critical threshold of PbI_2 and MACl concentrations leading to the formation of Cl compounds in the resulting film possibly such as Cl-doped perovskite ($FAPbI_{3-x}Cl_x$ where x is small) and Cl-based perovskite. This can be explained by the formation of an intermediate phase involving excess PbI_2 that prevents anion exchange, which results in $FAPbI_3$ with Cl^- remaining within the lattice even after thermal annealing. To probe the mechanism, the reaction dynamics relative to the excess PbI_2 should be considered:

1) Without excess PbI_2, the reaction in the intermediate state may proceed as follows:

   FAI + PbI_2 + MACl (55%) → MA, Cl-doped $FAPbI_3$ + unbound MACl + unbound FAI (replaced by the MACl doping).

   According to calculation in a previous study[11], the doping formation energy increases sharply when the MACl concentration exceeds 33%, which could result in some MACl remaining unbound. In the final state, due to the anion exchange where Cl^- doped in $FAPbI_3$ is exchanged by I^- from the unbound FAI, MA-doped $FAPbI_3$ remains, with other components evaporating during thermal annealing. Here, Cl^- primarily aids in lattice stabilisation[11].

2) However, with a substantial excess PbI_2 (e.g. ≥ 20%), the reaction becomes more complex:

   FAI + PbI_2 + MACl (55%) + excess PbI_2 → MA, Cl-doped $FAPbI_3$ + unbound MACl + unbound FAI (replaced by the MACl doping) + excess PbI_2.

   In this case, a portion of unbound FAI resulting from the MACl doping in $FAPbI_3$, along with the unbound MACl due to the doping limit, may react with the excess PbI_2, which is in the form of edge-sharing $[PbI_6]^{4-}$ octahedra. This reaction may lead to the formation of another intermediate phase, PbI_2-DMSO-(MACl + FAI). Thus, I^- from the unbound FAI becomes unavailable for anion exchange with Cl^-, which leads to the retention of Cl^- in $FAPbI_3$, promoting lattice contraction and the formation of face-sharing octahedra, ultimately driving the formation of the 6H polytype (Supplementary Fig. 5). The intermediate phase may subsequently transform into Cl-based compounds such as $MAPbCl_3$ or FA, I-doped $MAPbCl_3$ as observed in XRD (Supplementary Fig. 6)

3) When excess PbI_2 is limited (e.g. 10%), the intermediate phase primarily forms as PbI_2-DMSO-MACl without FAI. This occurs due to the stronger Pb–Cl bond relative to Pb–I bond[21,22], along with the limited availability of PbI_2 binding sites. As a result, I^- from the unbound FAI may undergo anion exchange, leading to de-doping of Cl^- from $FAPbI_3$. Therefore, lattice contraction is not promoted, and does not contribute to the formation of the 6H polytype.

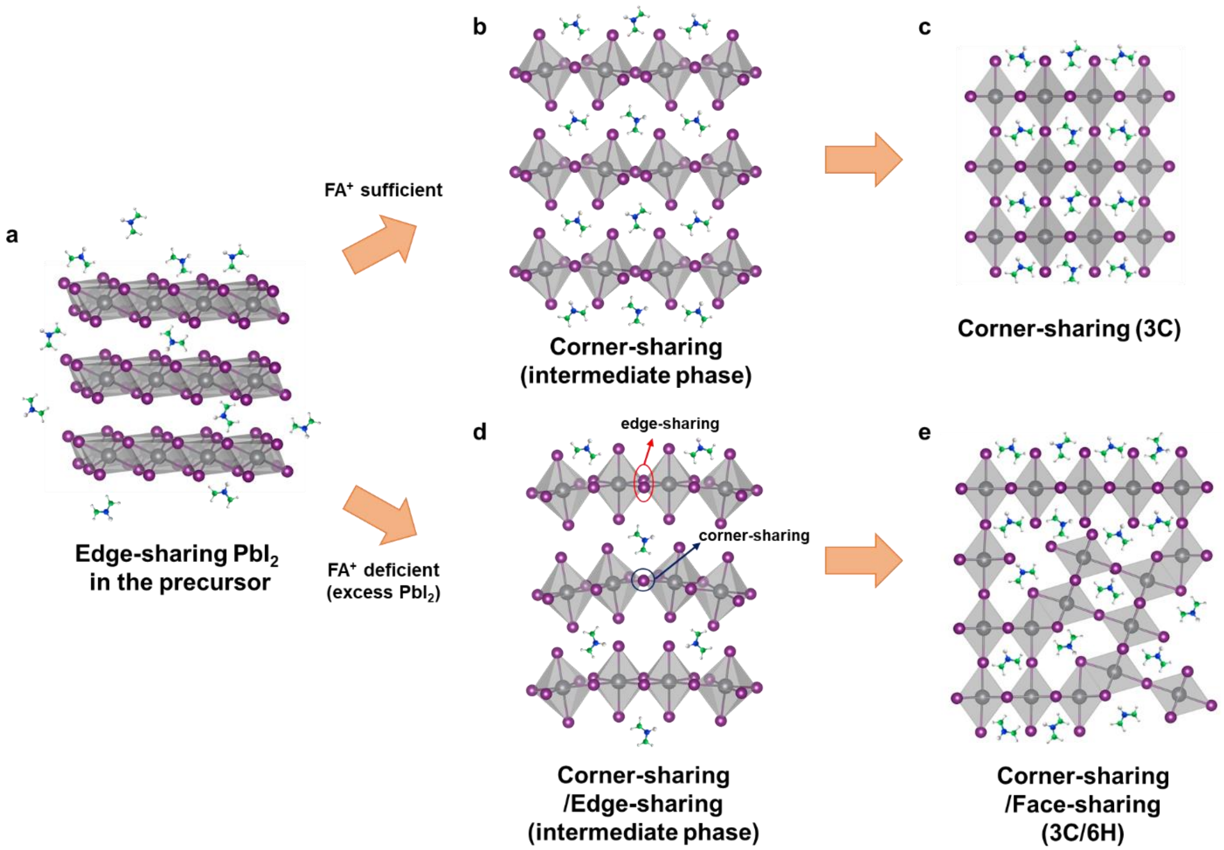

Supplementary Fig. 3.

Schematic of the proposed crystallization mechanism of the 3C/6H hetero-polytypic perovskite. **a**, edge-sharing PbI_2 in the precursor state. **b**, **c**, FA^+ intercalation leads to breakage of the edge connection and the formation of a corner-sharing intermediate phase, resulting in the 3C perovskite. **d**, **e**, Deficient FA^+ allows partial transformation of the edge-sharing PbI_2 into the corner-sharing intermediate phase, with the remaining edge-sharing octahedra sharing their faces to form the 3C/6H hetero-polytypic perovskite. The visualization was accomplished using VESTA[23].

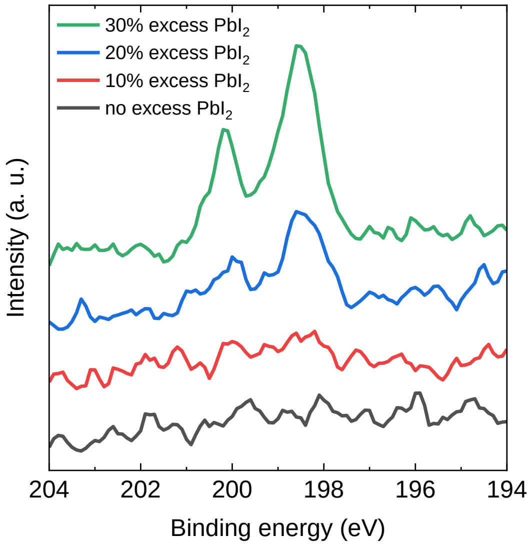

Supplementary Fig. 4.

Core level XPS of Cl 2*p* for the perovskite films varying the concentration of PbI_2 with a fixed concentration of MACl (55 mol%). The spectrum of the perovskite with 20% or 30% excess PbI_2 exhibits two peaks at the binding energy of 198.6 and 200.0 eV corresponding to Cl $2p_{3/2}$ and Cl $2p_{1/2}$ whereas the spectrum of the perovskite with 10% excess, or without excess PbI_2 does not contain any peaks.

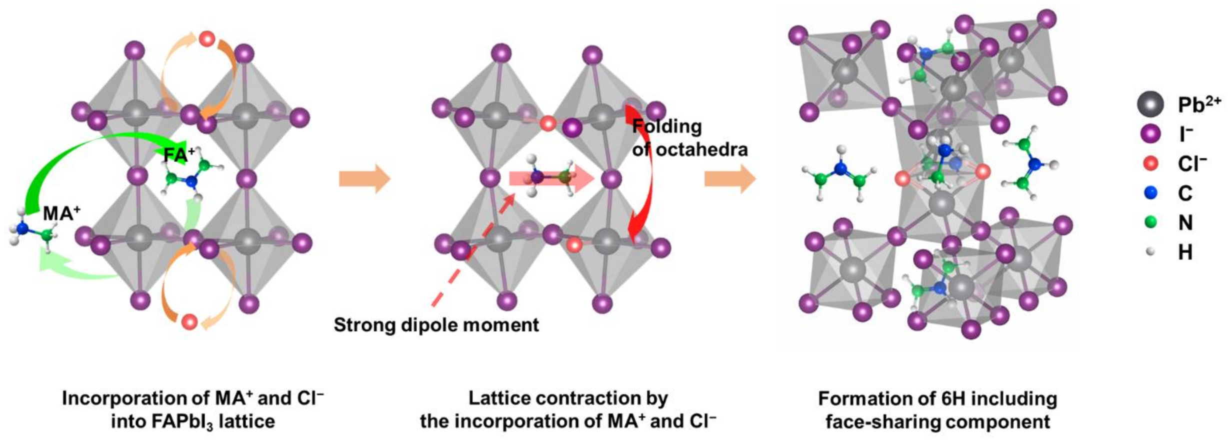

Supplementary Fig. 5.

Schematic of proposed mechanism for the formation of 6H polytype by incorporation of MA^+ and Cl^-. Doping MACl into $FAPbI_3$ results in lattice contraction due to the strong dipole moment of MA^+ and the smaller size of Cl^-. This presumably promotes folding of octahedra, leading to the formation of face-sharing component in the 6H polytype. The visualization was accomplished using VESTA[23].

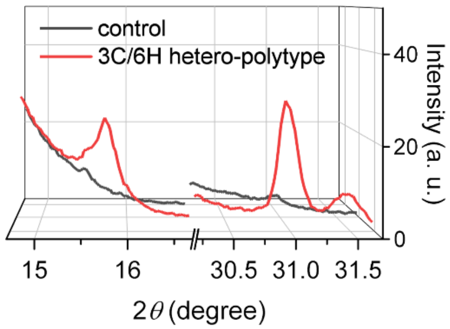

Supplementary Fig. 6.

Magnified XRD pattern of the control film and the 3C/6H hetero-polytypic perovskite film. The diffraction peaks at 15.7, 31.0, and 31.5° may be attributed to (100) and (200) of Cl-based perovskite (e.g. $MAPbCl_3$ or FA, I-doped $MAPbCl_3$).

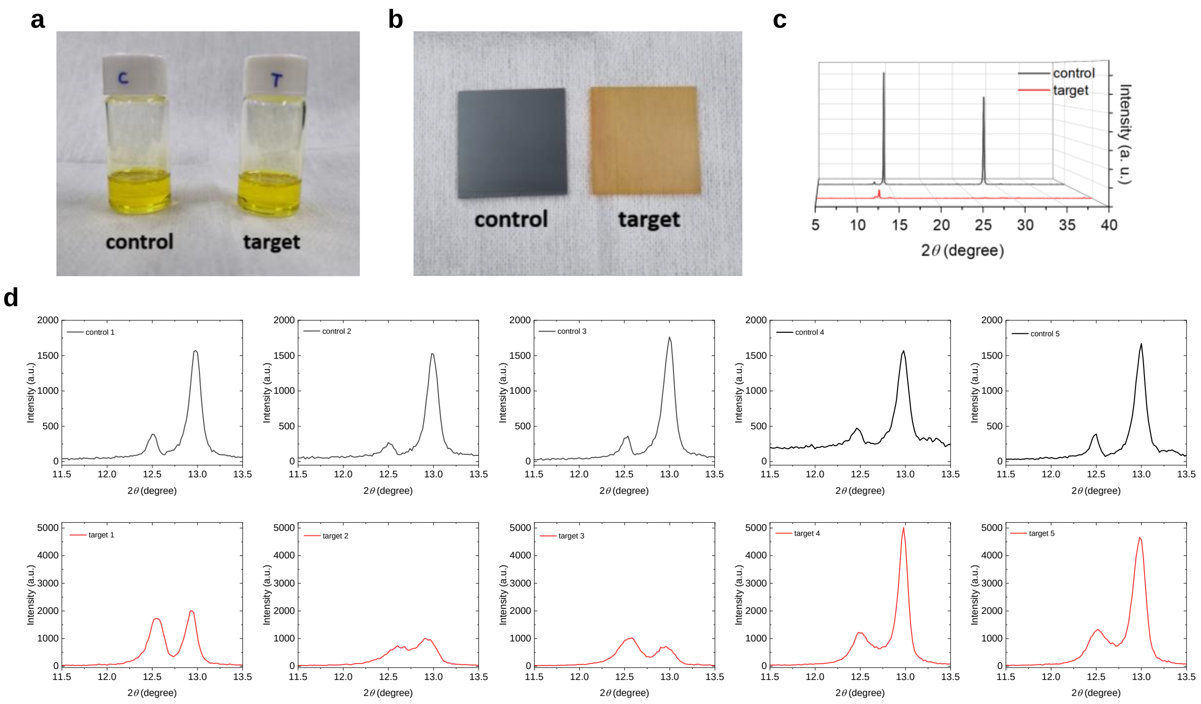

Supplementary Fig. 7.

a, Picture of the prepared solutions with excess PbI_2, control (left) and with $PbCl_2$ instead of excess PbI_2, target (right). **b**, The fabricated films using each precursor solution. **c**, The overall XRD patterns of the control and target perovskite films. **d**, XRD patterns of the perovskite films in the low 2θ angle region.

The target solution with $PbCl_2$ was prepared by dissolving FAI (1.2 M), PbI_2 (1.2 M), $PbCl_2$ (0.24 M), and MACl (0.66 M) in a mixed solvent comprising DMF:DMSO (8:1, v/v), whereas the control precursor solution with excess PbI_2 was prepared as described in the Materials and Methods: FAI (1.2 M), PbI_2 (1.44 M), and MACl (0.66 M) in DMF:DMSO (8:1, v/v). We confirmed that both solutions were well dissolved (Supplementary Fig. 7a).

After spin-coating and thermal annealing, the target film is orange in color, whereas the control film is black (Supplementary Fig. 7b). XRD analysis revealed that the target films have very low diffraction intensity of the 3C phase (Supplementary Fig. 7c), implying significant thermodynamic changes due to the altered composition. This variation may also lead to low reproducibility, with diffraction patterns showing inconsistent peaks for 6H and PbI_2 (Supplementary Fig. 7d). In contrast, the control films are highly reproducible, showing consistent diffraction patterns for 6H and PbI_2. The presence of the PbI_2 peak in the target films, despite not using excess PbI_2, suggests that PbI_2 does not fully react to form the 3C phase and remains as a residue.

Supplementary Note 3

The α-$FAPbI_3$ (3C) phase has a tendency to spontaneously transform into the δ phase (2H) under certain conditions, such as exposure to a liquid interface (e.g. water) or the atmosphere at room temperature[24,25]. Thus, in the case without the 6H phase, it is also likely that the formation of the 2H phase may be attributed to the phase transition by the exposure of the α-$FAPbI_3$ to air containing moisture. In contrast, in the presence of the 6H phase, the phase transformation of $FAPbI_3$ from the α phase to the δ phase is effectively prevented, as shown in Fig. 1b and 1d. In order to gain insights into the contribution of the 6H phase towards stabilizing the 3C α phase, Born-Oppenheimer molecular dynamics simulations were performed to assess the effect of temperature on the stability of 2H, 6H and 3C phases. The thermodynamics of the 2H, 6H, and 3C phases were analysed at temperatures of 300 and 423 K (Supplementary Table 3). It has been reported that rotation of FA^+ cations provides the major contribution[26,27] to the difference of the finite temperature entropy contribution between the δ-$FAPbI_3$ (2H) phase and the α-$FAPbI_3$ (3C) phase. Therefore, the rotation of the FA^+ entropically stabilizes the 6H and 3C phases, resulting in a substantial barrier for the conversion of the 3C phase into the 2H phase (Supplementary Fig. 8).

Supplementary Table 3.

Calculated thermodynamic energy difference among the 2H, 6H, and 3C phases at 300 and 423 K.

**at 300 K**			**at 423 K**		
ΔU_{6H-2H}, eV	ΔU_{2H-3C}, eV	ΔU_{6H-3C}, eV	ΔU_{6H-2H}, eV	ΔU_{2H-3C}, eV	ΔU_{6H-3C}, eV
0.11	0.17	0.06	0.08	0.14	0.06
ΔF_{6H-2H}, eV	ΔF_{3C-2H}, eV	ΔF_{3C-6H}, eV	ΔF_{6H-2H}, eV	ΔF_{3C-2H}, eV	ΔF_{3C-6H}, eV
-0.17	-0.11	0.06	-0.34	-0.28	0.06
ΔS_{6H-2H}, eV	ΔS_{3C-2H}, eV	ΔS_{3C-6H}, eV	ΔS_{6H-2H}, eV	ΔS_{3C-2H}, eV	ΔS_{3C-6H}, eV
-0.28	-0.28	0.00	-0.42	-0.42	0.00

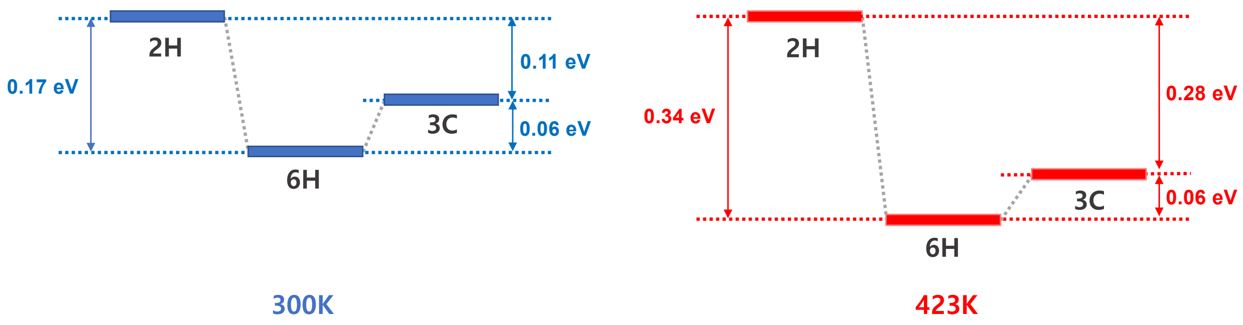

Supplementary Fig. 8.

Thermodynamic energy diagrams that show the energy differences among 2H, 6H, and 3C-$FAPbI_3$ at 300 and 423 K. The Helmholtz free energy differences in eV for the 2H, 6H and 3C phases are presented.

<u>Computational details</u>

Simulations were performed in the framework of density functional theory (DFT). The initial structures of 3C-$FAPbI_3$ (4 × 4 × 4 with random orientation of FA), 2H-$FAPbI_3$ and 6H-$FAPbI_3$ were fully optimized both in terms of atomic positions and cell parameters within the density functional theory using the Perdew-Burke-Ernzerhof (PBE)[28] functional, localized atomic DZVP-MOLOPT basis set[29] for the wavefunction expansion, plane wave auxiliary basis set with 600 Ry cutoff for the charge density representation and norm-conserving Goedecker-Teter-Hutter[30] (GHT) pseudopotentials for the description of core-valence region. The (110) surfaces were cut for both of these phases. To simulate the localized polaron due to the positively charged iodine vacancy, the (110) surface of 3C-$FAPbI_3$ phase having V_I^+ defect was fully relaxed using hybrid PBE0 density functional[31,32] and Auxiliary-Density-Matrix-Method (ADMM)[33] used to accelerate the PBE0 functional with cFIT9, cFIT7 and cFIT3 basis sets for Pb, I, and C/N/H atoms, respectively. Due to the large extent of the simulated unit cells, the reciprocal space was sampled at the Γ-point in all cases.

To account for the temperature effect on the stability of the 2H, 6H and 3C phases, Born-Oppenheimer molecular dynamics (BOMD) simulations were performed for several picoseconds for each phase using the timestep of 1 fs and maintaining the temperature constant at 300 and 423 K, employing the velocity rescaling algorithm. The corresponding internal energies were extracted following a previously reported procedure[34]. For the 6H and 3C phases a free rotation of the FA^+

cations was assumed to give the major contribution of the entropy difference at finite temperatures with the 2H phase, in which this rotation is blocked, as reported previously[26,27]. The rotational entropy due to the FA^+ cation was computed using the formula, derived from statistical thermodynamics considerations:

$$S_{rot} = \frac{3}{2}k\left[1 + \ln\left(\frac{2\sqrt[3]{\pi}}{\hbar^2}kT\sqrt[3]{I_1 I_2 I_3}\right)\right] \quad (1)$$

where I_1 = 8.586 uÅ^2, I_2 = 48.851 uÅ^2, I_3 = 57.436 uÅ^2.[2] All the calculations are performed with CP2K code[35]. The visualization was accomplished using VESTA[23].

Supplementary Note 4

ImageJ (https://imagej.nih.gov/ij/), a free software for image processing developed at National Institutes of Health (NIH), was used to determine the grain size of perovskite and the occupation rate of PbI_2 on the surface of perovskite films.

1) Determination of grain size

A straight line was drawn on a SEM surface image and the number of grain boundaries which intersect the line was counted. The average grain size was obtained by dividing the line length given the scale by the number of intersections.

2) Estimation of the occupation rate of PbI_2 on perovskite surface.

As PbI_2 grains are shown brighter than perovskite grains in SEM surface image, heightening the contrast makes it easier to differentiate those two different grains. To adjust the color contrast, the Threshold command was applied via the Image>Adjust>Threshold submenu. Occupation rate of darker grains was collected using the "Area fraction" function.

Supplementary Note 5

The energy of the iodine vacancy formation with the charge q = +1, 0, –1 is computed as follows:

$$\Delta_f E_{vac^q} = E_{surf+vac^q} - E_{surf} + \mu_I + q(\varepsilon_F + \varepsilon_{VBM}) \quad (2)$$

where $E_{surf+vac^q}$ and E_{surf} are the energies of the defective and pristine (110) surface of 3C-$FAPbI_3$, μ_I is the chemical potential of iodine with the atomic energy of iodine taken as a reference, ε_F is the Fermi level defined with respect to the energy of the valence band maximum ε_{VBM}.

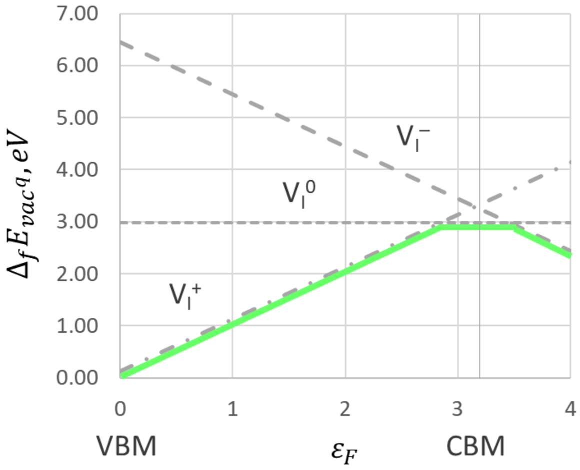

Supplementary Fig. 9.

Formation energies of iodine vacancies on (110) 3C-$FAPbI_3$ surface, computed at PBE0 level, taking the energy of atomic I as a reference for μ_I.

Supplementary Table 4.

Summary of the formation energies of iodine vacancies on (110) 3C-$FAPbI_3$ surface in the Supplementary Fig. 9.

Type of vacancy	$\Delta_f E_{vac^q}$ (eV)
V_I^+	0.13
V_I^0	2.98
V_I^-	6.45

Supplementary Note 6

Charge transition energy levels from a state with a charge q to a state with a charge q' are defined as follows with respect to the valence band maximum ε_{VBM}, taken as a reference energy:

$$E(q/q') = \frac{\Delta_f E_{vac^q} - \Delta_f E_{vac^{q'}}}{q' - q} = \frac{E_{vac\_q} - E_{vac\_q'}}{q' - q} - \varepsilon_{VBM} \qquad (3)$$

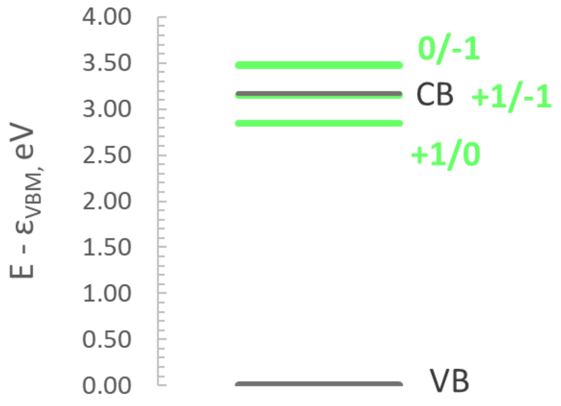

Supplementary Fig. 10.

Charge transition energy levels for iodine vacancies on (110) 3C-$FAPbI_3$ surface, computed using PBE0 density functional with respect to ε_{VBM} ($E(q/q')$).

Supplementary Table 5.

Summary of the charge transition energy levels for iodine vacancies on (110) 3C-$FAPbI_3$ surface in the Supplementary Fig. 10.

Transition for V_I	$\boldsymbol{E(q/q')}$ (eV)
+1/0	2.84
0/-1	3.47
+1/-1	3.16

Supplementary Note 7

Elliott's theory was implemented to deconvolute absorbance to reveal contribution from excitonic resonance and from continuum state, which can be expressed as:

$$\alpha(E) \propto \frac{\mu_{cv}^2}{E}\left[\sum_n \frac{4\pi\sqrt{E_b{}^3}}{n^3}\delta\left(E-\left(E_g-\frac{E_b}{n^2}\right)\right)+\frac{2\pi\sqrt{\frac{E_b}{E-E_g}}\theta}{1-e^{-2\pi\sqrt{\frac{E_b}{E-E_g}}}}\right] \quad (4)$$

where μ_{cv} is transition dipole moment, E_b is the exciton binding energy, n is the principal quantum number of the exciton state, δ is the Dirac delta function, θ is the step function. The first term accounts for the contribution from the excitonic resonance while the second term reflects the contribution from the continuum state.

Supplementary Note 8

The effective lifetime of charge carriers is a function of radiative recombination rate, and phonon-assisted non-radiative recombination rate[36], which comprises both band-to-band phonon-assisted transitions and so-called trap-assisted recombination (Shockley-Read-Hall recombination):

$$\frac{1}{\tau} = \frac{1}{\tau_r} + \frac{1}{\tau_{nr}} + \frac{1}{\tau_{SRH}} \tag{5}$$

The last term (τ_{SRH}) can greatly accelerate electron-hole recombination and is dependent on the density of trap states (n_t) and their energy, as well as the probability of transitions between excited and ground electronic states. The deep trap states originating from the reduction of V_I^+ facilitate the electron-hole recombination serving as intermediate states for charge carrier accumulation and reducing the effective band gap between the occupied and empty electronic states in perovskites. Assuming accumulation of the deep traps at the 3C-$FAPbI_3$ facets, the ratio between carrier lifetime is proportional to the ratio of trap state concentration (n_t) and the wavefunction overlaps between electrons and holes:

$$\frac{\tau_{3C/6H}}{\tau_{3C}} \sim \frac{n_{t3C}}{n_{t3C/6H}} \cdot \frac{\langle \Psi_h | \Psi_e \rangle_{3C}}{\langle \Psi_h | \Psi_e \rangle_{3C/6H}} \tag{6}$$

since the non-adiabatic coupling coefficients, determining the rate of non-radiative recombination, correlate, among others, with the spatial overlap of the hole and electron wavefunctions[36]. In practice, the trap density in the hetero-polytypic 3C/6H perovskite is decreased by a factor of 2.12 as compared to the 3C phase. Besides, the 6H phase bridging enables an increase in the linear size of the grain by ×2.17 (Supplementary Table 7), thus increasing the grain volume by an order of magnitude. This, in its turn, extends the space, within which the charge carriers can be separated, by an order of magnitude as well. Thus, the incorporation of the 6H phase is favorable for the charge separation; consequently, it decreases the wavefunction overlap and increases the charge carrier lifetime. The theoretically predicted lifetime extension by ×22 assuming cubic grains and by ×28 assuming spherical grains, leading on average to a ×25 enhancement, is consistent with the practically observed increase in the average lifetime by a factor of 25 from 0.27 μs to 6.73 μs.

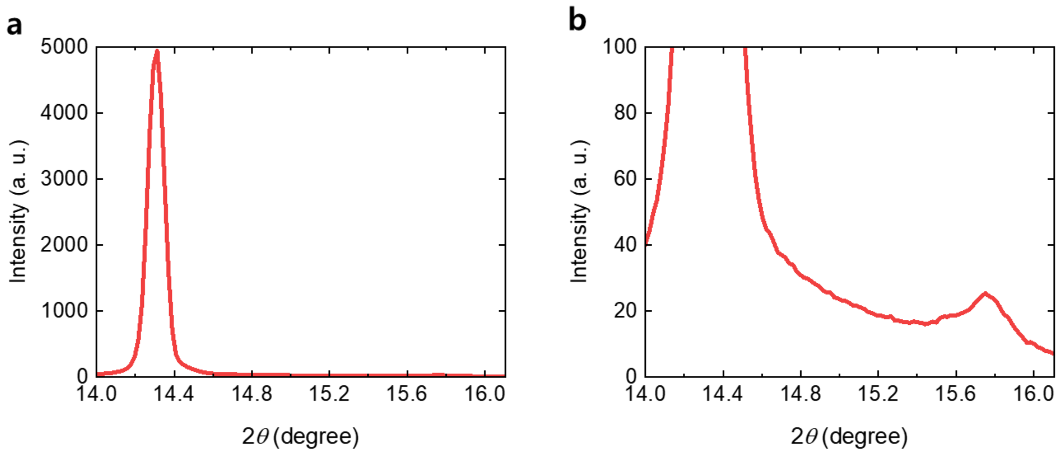

Supplementary Fig. 11.

a, XRD pattern of the 3C/6H hetero-polytypic perovskite film showing the diffraction of (100) of $FAPbI_3$ with an intensity of 4908. **b**, The same pattern with 50 times magnified intensity showing the diffraction of (100) of $MAPbCl_3$ with an intensity of 25.

Supplementary Table 6.

Crystallographic details of the polytypes detected in the $FAPbI_3$ perovskite single crystals.

Polytype	3C	2H	6H
Formula	$CH_5N_2PbI_3$	$CH_5N_2PbI_3$	$CH_5N_2PbI_3$
$D_{calc.}$/ g cm^{-3}	4.153	3.794	3.852
μ/mm^{-1}	25.697	24.233	25.686
Formula Weight	632.98	632.98	632.98
Colour	metallic dark black	clear light yellow	clear intense orange
Shape	prism-shaped	prism-shaped	prism-shaped
Size/mm^3	0.11×0.08×0.03	0.09×0.07×0.05	0.20×0.11×0.06
T/K	291.99(10)	294.02(10)	292.8(3)
Crystal System	cubic	hexagonal	hexagonal
Space Group	$Pm\bar{3}m$	$P6_3/mmc$	$P6_3/mmc$
a/Å	6.3290(2)	8.7904(11)	8.8424(15)
b/Å	6.3290(2)	8.7904(11)	8.8424(15)
c/Å	6.3290(2)	8.0314(11)	22.454(5)
α/°	90	90	90
β/°	90	90	90
γ/°	90	120	120
V/Å^3	253.51(2)	537.45(15)	1520.4(6)
Z	1	2	6
Z'	0.020833	0.083333	0.25
Wavelength/Å	0.71073	0.71073	0.71073
Radiation type	Mo$K\alpha$	Mo$K\alpha$	Mo$K\alpha$
Θ_{min}/°	3.219	3.688	3.220
Θ_{max}/°	32.386	29.510	32.690
Min. / Max. h k l	$-8 \leq h \leq 9$<br>$-9 \leq k \leq 5$<br>$-9 \leq l \leq 9$	$-10 \leq h \leq 8$<br>$-7 \leq k \leq 11$<br>$-9 \leq l \leq 10$	$-11 \leq h \leq 13$<br>$-13 \leq k \leq 11$<br>$-16 \leq l \leq 34$
Measured Refl's.	1633	1571	5427
Indep't Refl's	125	293	1037
Refl's I≥2σ(I)	120	178	594
R_{int}	0.0241	0.0440	0.0490
Absorption Coefficient / mm^{-1}	25.697	24.233	25.686
Parameters	8	14	16
Restraints	1	7	0
Largest Peak/e Å^{-3}	0.638	1.062	3.998
Deepest Hole/e Å^{-3}	-0.891	-0.840	-2.545
GooF	1.124	1.132	1.051
wR_2 (all data)	0.0532	0.1336	0.3275
wR_2	0.0529	0.1189	0.2942
R_1 (all data)	0.0203	0.0821	0.1136
R_1	0.0194	0.0438	0.0819
CCDC number	2115270	2115268	2115269

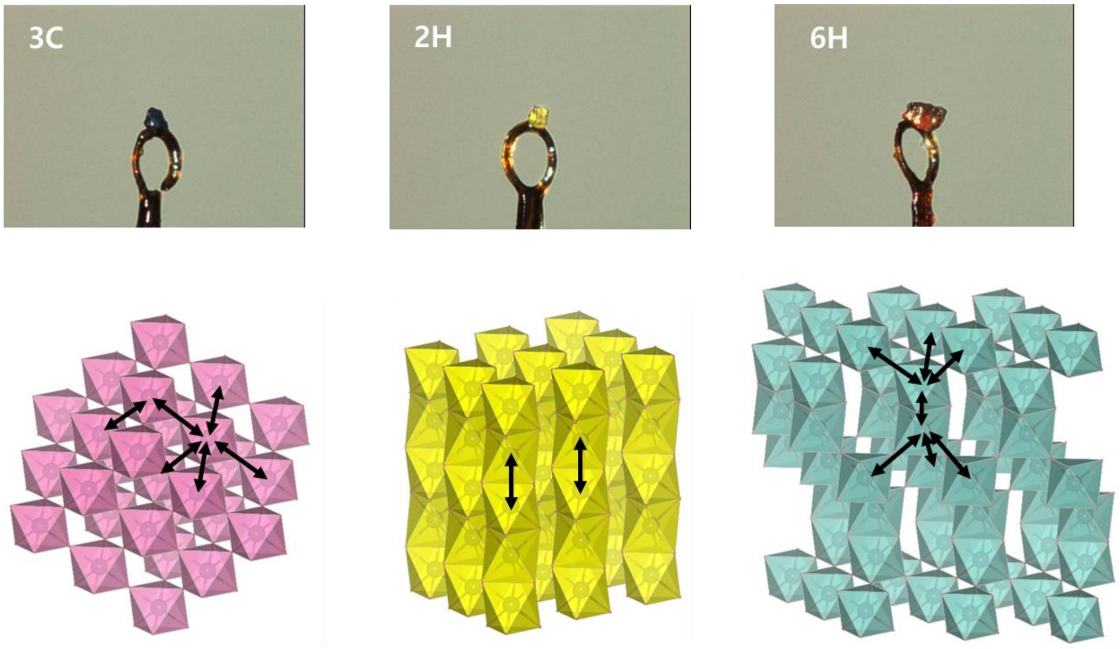

Supplementary Fig. 12.

Single crystals of 3C, 2H, and 6H for SCXRD measurement (top row). Supercells of 3 × 3 × 3 3C-$FAPbI_3$, 2 × 2 × 2 2H-$FAPbI_3$, and 3 × 2 × 1 6H-$FAPbI_3$ (bottom row). Black arrows indicate the possible paths of efficient carrier hopping. The visualization was accomplished using VESTA[23].

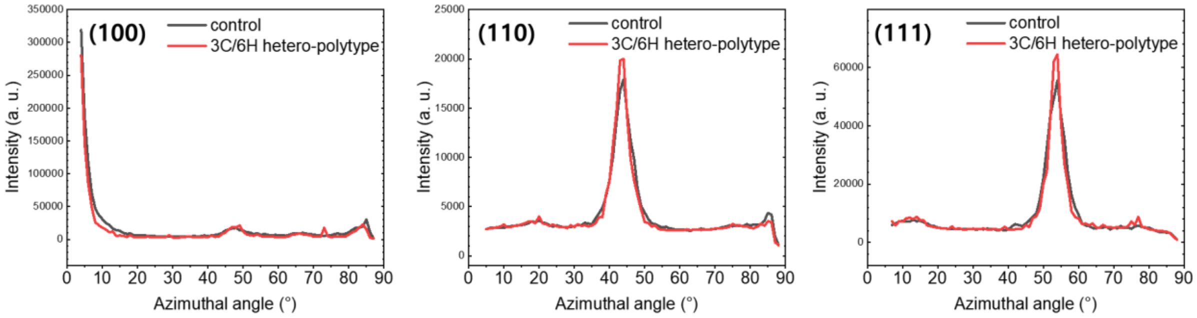

Supplementary Fig. 13.

The azimuthal angle-dependent diffraction intensity of (100), (110), and (111) of the control film and the 3C/6H hetero-polytypic perovskite film. The diffraction of the planes was integrated along the scattering vector between 0.95~1.05 $Å^{-1}$ for (100), 1.35~1.45 $Å^{-1}$ for (110), and 1.67~1.75 $Å^{-1}$ for (111). Both films show a similar pattern of diffraction of the planes; (100) exhibited an explicitly strong out-of-plane diffraction although signals were only detectable from 4°, and (110) and (111) resulted in the peak intensity at 44° and 54°. This indicates that the perovskites are nearly uniaxial with the highly ordered crystalline structure.

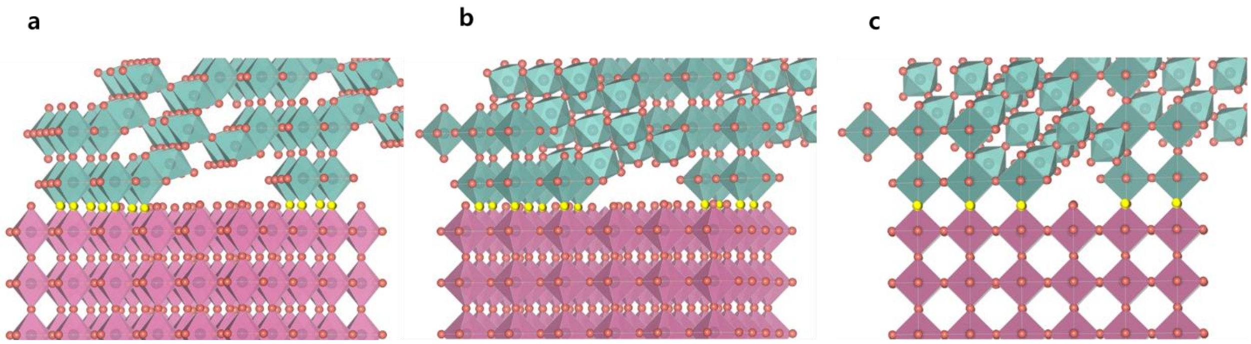

Supplementary Fig. 14.

3D lattice arrays exhibiting the regional site of the blue boxed area in Fig. 1f in which 3C and 6H phases are connected based on the intervened corner-sharing halides that result in the high coherency between two phases at the phase boundary. The array was rotated by (**a**) 10°, (**b**) 30° and (**c**) 45° along the y-axis. The visualization was accomplished using VESTA[23].

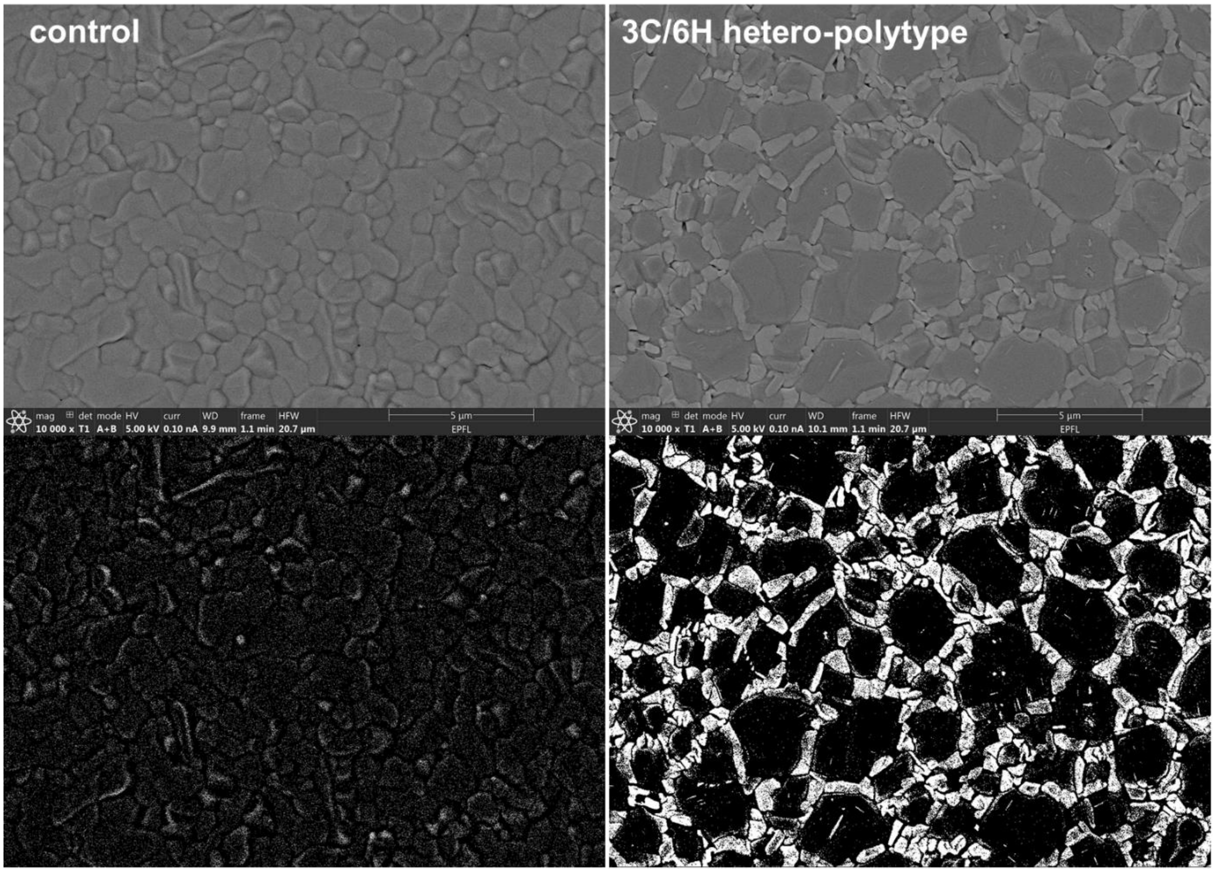

Supplementary Fig. 15.

SEM images of surface of the control film and the 3C/6H hetero-polytypic perovskite film (top row), and the same images but with high contrast to explicitly differentiate PbI_2 and perovskite grain (bottom row). The control film consisted of homogeneous grains, whereas the film with the 6H polytype exhibited brighter grains intercalated between the darker perovskite grains. The brighter grains can be attributed to the excess PbI_2 that occupied 24.8% of the surface according to the occupation rate obtained by the use of software ImageJ.

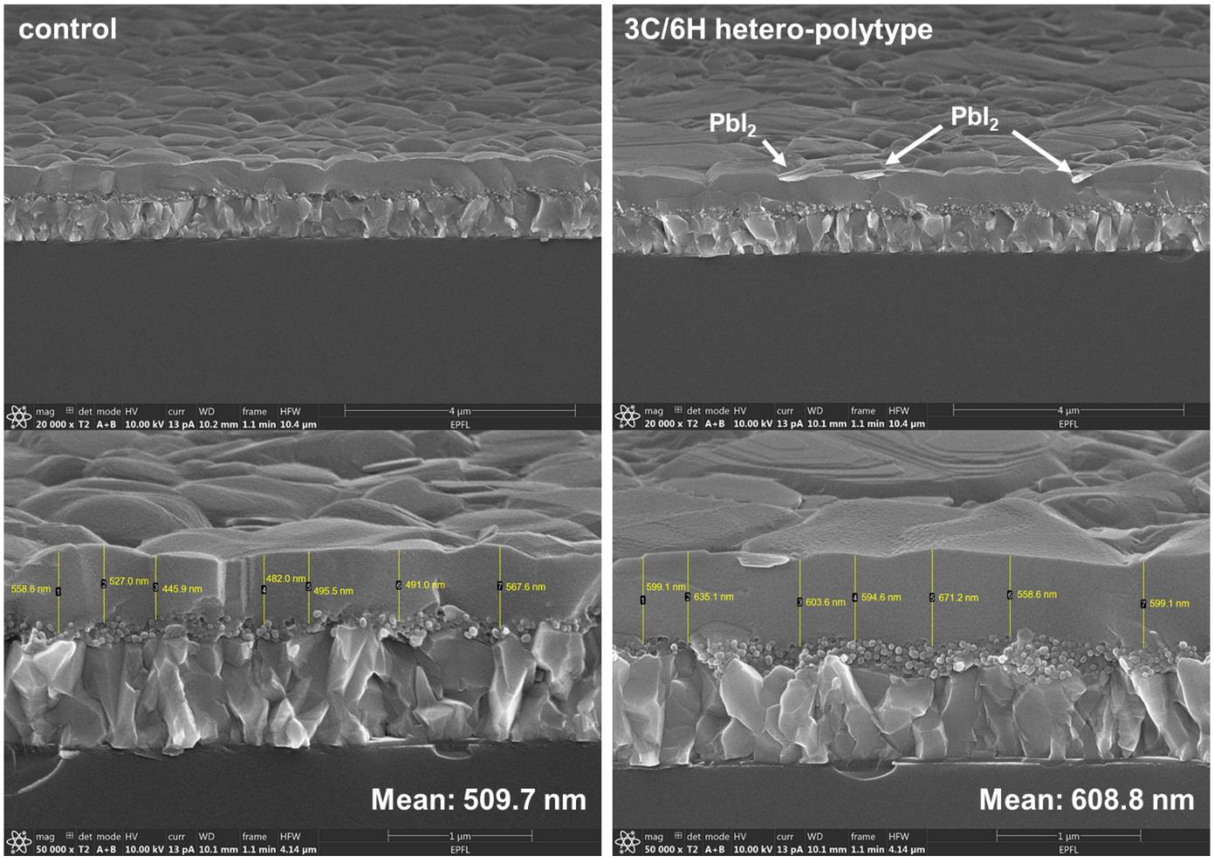

Supplementary Fig. 16.

Cross-sectional SEM images of the control perovskite film (left) and the 3C/6H hetero-polytypic perovskite film (right) having mean thickness values of 509.7 and 608.8 nm, respectively.

Supplementary Table 7.

Average grain size of the control perovskite and the 3C/6H hetero-polytypic perovskite.

	Average grain size (μm)
Control	0.762
3C/6H hetero-polytype	1.654

Supplementary Table 8.

Fitting result of PL decay curves of perovskite films by a bi-exponential decay function, $y = A_1 e^{-t/\tau_1} + A_2 e^{-t/\tau_2}$ where τ_1 and τ_2 are the lifetimes for the fast and slow decays.

	A_1	τ_1 (μs)	A_2	τ_2 (μs)	τ_{ave} (μs)
Control	0.157	0.04	0.783	0.28	0.27
3C/6H hetero-polytype	0.300	2.08	0.518	7.48	6.73
3C/6H hetero-polytype w/ LDP	0.308	10.51	0.317	22.74	18.95

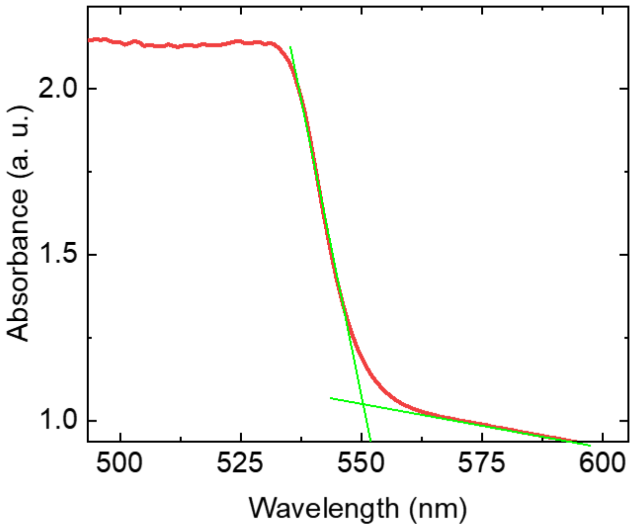

Supplementary Fig. 17.

Magnified version of absorbance in Fig. 3a exhibiting secondary absorption onset at 550 nm by the 6H polytype.

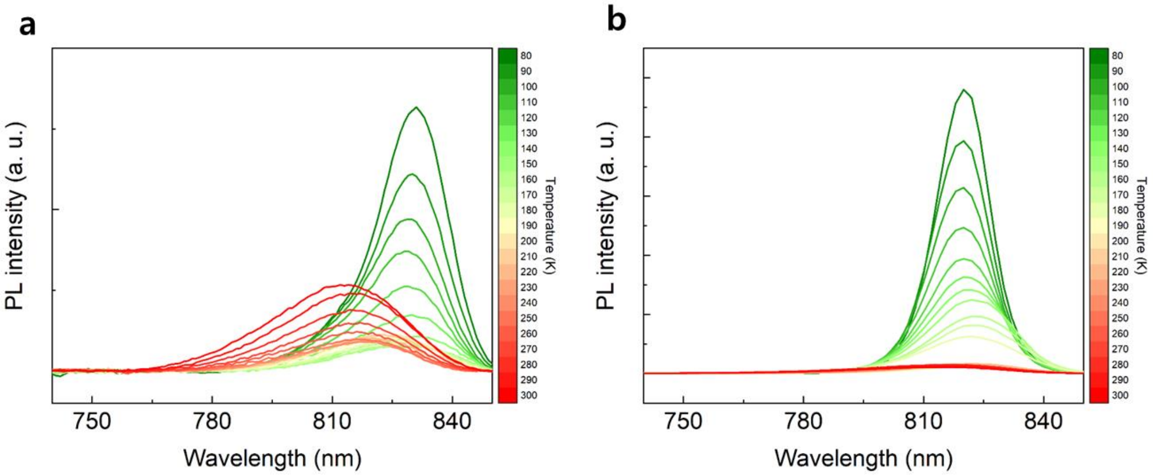

Supplementary Fig. 18.

Temperature-dependent PL spectra of (**a**) the control film and (**b**) the 3C/6H hetero-polytypic perovskite film.

Supplementary Table 9.

In metal halide perovskites, as shown elsewhere, electron-phonon coupling is predominantly governed by Fröhlich interaction of the longitudinal optical (LO) mode (Γ_{LO}) in addition to disorder-induced scattering that results in temperature-independent inhomogeneous PL broadening (Γ_0)[37]. Thus, the summation of the contributions to the temperature-dependent PL linewidth can be expressed as:

$$\Gamma(T) = \Gamma_0 + \Gamma_{ac} + \Gamma_{LO} + \Gamma_{imp} = \Gamma_0 + \frac{\gamma_{LO}}{e^{\frac{E_{LO}}{k_B T}} - 1} \quad (7)$$

in which γ_{LO} is the coupling strength between electrons and LO phonons and E_{LO} is the representative energy of LO phonons described by the Bose-Einstein distribution function. Linewidth broadening parameters were extracted from the temperature-dependent PL measurements of the control perovskite film and the 3C/6H hetero-polytypic perovskite film. Γ_0: temperature-independent inhomogeneous PL broadening, E_{LO}: representative energy of longitudinal optical (LO) phonon, and γ_{LO}: electron-LO phonon coupling strength.

	Γ_0 (meV)	E_{LO} (meV)	γ_{LO} (meV)
Control	40.78	33.37	74.84
3C/6H hetero-polytype	24.64	14.62	41.31

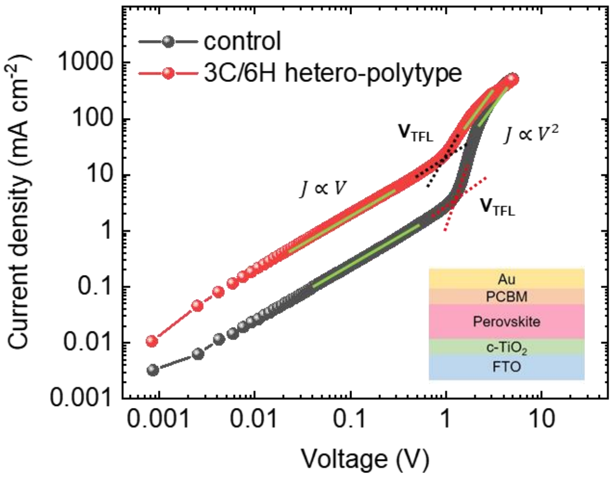

Supplementary Fig. 19.

To verify the difference in the carrier mobility, electron-only devices (EODs) using the control perovskite film (black) and the 3C/6H hetero-polytypic perovskite film (red) were employed with a structure composed of FTO/compact-TiO_2/perovskite/PCBM/Au. Electron mobility (μ_e) of the perovskites was estimated by fitting the space-charge-limited current (SCLC) of the devices to the Mott–Gurney equation, $J = \frac{9\varepsilon\varepsilon_0\mu_e V^2}{8L^3}$ where J is dark current density of a device, V is applied voltage, L is the thickness of perovskite, ε is the relative dielectric constant (here, we used 46.9), and ε_0 is the vacuum permittivity[38,39]. The μ_e of the control film is 0.697 $cm^2\ V^{-1}\ s^{-1}$, and the incorporation of the 6H polytype significantly increases the mobility to 1.894 $cm^2\ V^{-1}\ s^{-1}$.

Besides, the trap density (n_t) of the perovskites was estimated according to the following equation, $V_{TFL} = n_t \frac{eL^2}{2\varepsilon\varepsilon_0}$ where e is the elementary charge of the electron[40]. V_{TFL} is defined a point where a transition of J–V behavior from Ohmic to the trap-filling limited (TFL) regime occurs. The EOD with the control perovskite has a V_{TFL} of 1.27 V, and the device with the 6H polytype had a V_{TFL} of 0.93 V. For the control film $n_t = 2.48 \times 10^{16}$ cm^{-3}, and for the film with the 6H phase $n_t = 1.17 \times 10^{16}$ cm^{-3}, indicating that the 6H phase effectively passivates the defects within the perovskite.

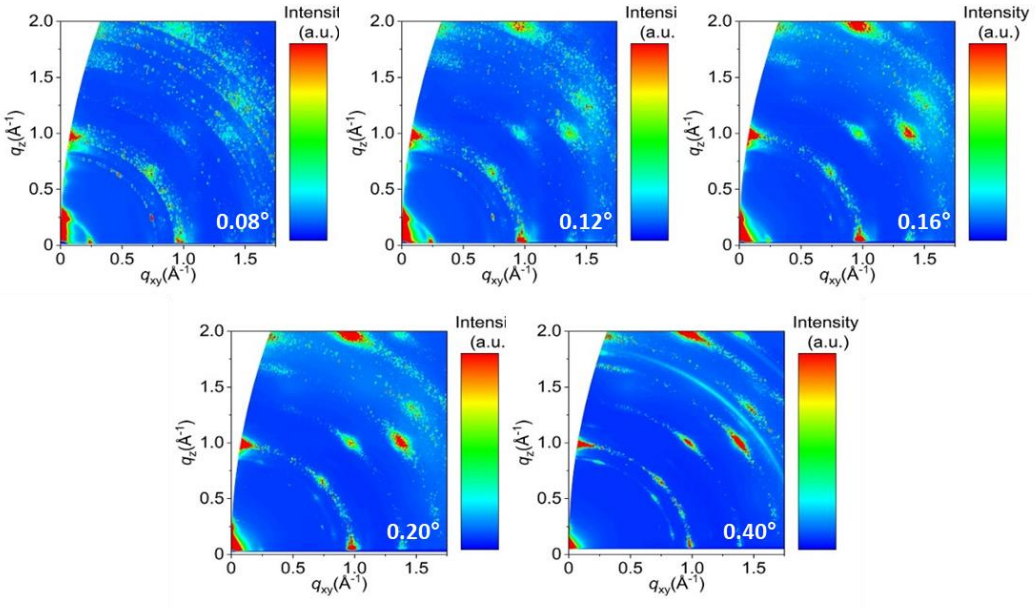

Supplementary Fig. 20.

GIWAX patterns of the 3C/6H hetero-polytypic perovskite film with a surface passivation layer on top. The X-ray incident angle was varied from 0.08° to 0.40°. When the X-ray incident angle was lower than 0.40°, a new diffraction pattern with a scattering vector of q ~ 0.24 Å^{-1} explicitly appeared, which indicates the formation of low-dimensional perovskite (LDP).

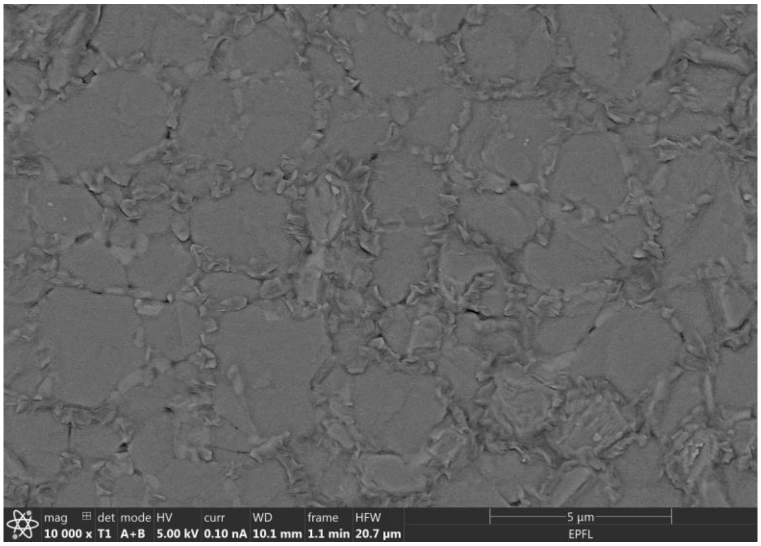

Supplementary Fig. 21.

SEM image of the 3C/6H hetero-polytypic perovskite film with surface passivation by LDP.

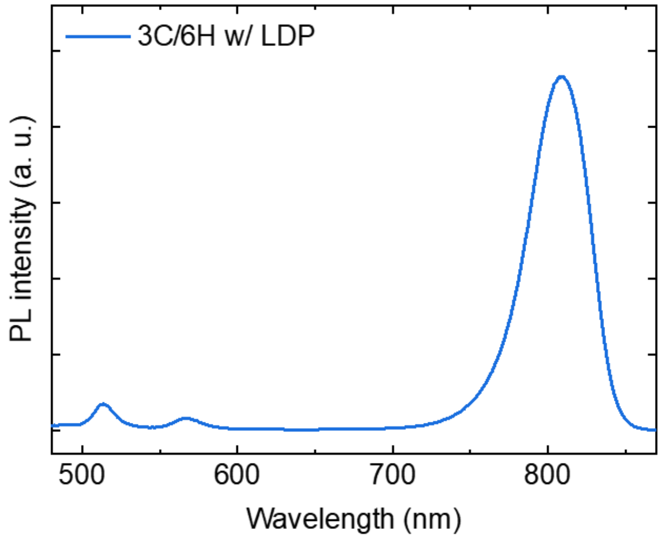

Supplementary Fig. 22.

PL spectrum of the 3C/6H hetero-polytypic perovskite film with surface passivation by LDP.

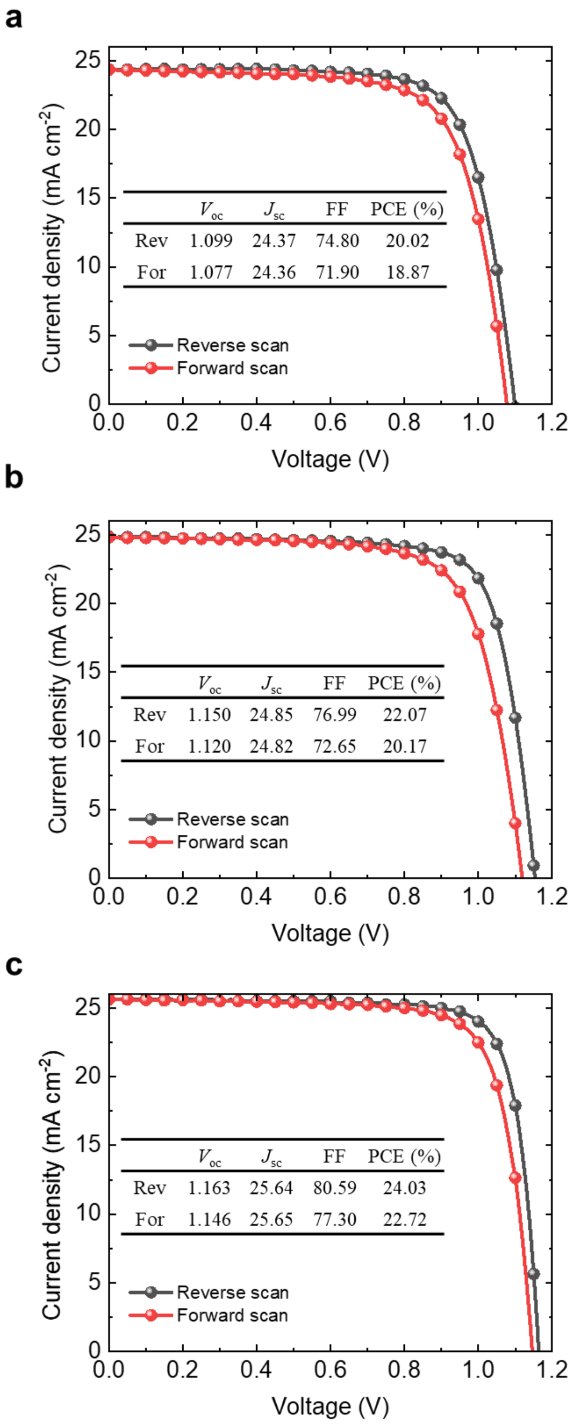

Supplementary Fig. 23.

J-*V* curves of PSCs using (**a**) the control perovskite, (**b**) the 3C/6H hetero-polytypic perovskite, and (**c**) the 3C/6H hetero-polytypic perovskite with surface passivation under reverse and forward scan.

Supplementary Table 10.

J-V characteristics of PSCs reported in the device performance statistics in Fig. 5a.

	V_{oc} (V)	J_{sc} (mA/cm^2)	FF	PCE (%)
**Control**	1.083	24.50	73.98	19.62
	1.080	24.16	74.35	19.39
	1.091	24.49	74.61	19.93
	1.079	23.79	74.71	19.17
	1.091	24.31	75.79	20.09
	1.100	24.24	74.32	19.81
	1.098	24.40	75.82	20.32
	1.092	23.68	76.49	19.78
	1.096	24.45	75.62	20.26
	1.102	24.47	72.58	19.57
	1.091	24.28	75.18	19.92
	1.087	23.89	75.36	19.57
	1.088	24.29	75.28	19.88
	1.086	23.89	73.74	19.13
	1.063	24.06	74.19	18.97
	1.060	23.76	73.74	18.58
	1.069	24.01	75.47	19.37
	1.072	24.12	74.95	19.37
	1.066	23.97	74.18	18.96
	1.059	23.71	74.31	18.66
**3C/6H hetero-polytype**	1.125	24.69	76.7	21.30
	1.121	24.83	77.37	21.53
	1.129	24.51	77.36	21.40
	1.131	24.75	76.95	21.54
	1.130	24.64	77.71	21.63
	1.132	24.33	76.93	21.18
	1.139	24.37	76.63	21.26
	1.141	24.71	77.61	21.88
	1.147	24.67	76.38	21.60
	1.149	24.75	74.69	21.23
	1.150	24.94	75.56	21.66
	1.146	24.93	76.01	21.71
	1.144	24.75	76.69	21.72
	1.144	24.78	74.88	21.23
	1.140	24.91	77.43	22.06
	1.150	24.85	76.99	22.07
	1.150	24.64	77.54	22.01
	1.140	24.80	78.72	22.35
	1.160	25.01	75.94	22.07
	1.160	24.96	77.08	22.31
**3C/6H**	1.164	25.69	80.22	23.99
	1.154	25.36	80.06	23.44

**hetero-polytype w/ LDP**	1.163	25.07	80.03	23.33
	1.163	25.64	80.59	24.03
	1.150	25.43	79.47	23.25
	1.154	25.46	79.83	23.46
	1.157	25.23	79.58	23.24
	1.156	25.58	81.60	24.13
	1.163	25.20	79.11	23.18
	1.150	25.61	79.33	23.37
	1.165	25.60	79.22	23.63
	1.160	25.48	78.76	23.29
	1.163	25.26	79.01	23.22
	1.165	25.44	78.63	23.30
	1.166	25.49	79.27	23.56
	1.166	25.34	80.09	23.66
	1.170	25.31	80.15	23.74
	1.170	25.39	80.06	23.79
	1.174	25.62	79.21	23.81
	1.155	25.76	80.14	23.85

KIER

Test Results

Report No. : KIER-211221012
Page(2)/(4)Pages

KOLAS — KOREA LABORATORY ACCREDITATION SCHEME — TESTING NO.KT201

Test result of perovskite solar cell		
Client	Ecole Polytechnique Federale de Lausanne (EPFL)	
Test item	Solar-cell materials and devices	
Detailed test item	I-V characteristic curve measurement	
Test condition	AM1.5G, 100 mW/cm^2, (25.0 ± 1.0) ℃	
Test method used	KS C IEC60904-1:2009<br>(6. Measurement in steady-state simulated sunlight [method C])	
Data corrections	Temperature & irradiance corrections (KS C IEC 60891)	(☑ applied, ☐ unapplied)
	Spectral mismatch correction (KS C IEC 60904-7)	(☐ applied, ☑ unapplied)<br>(MMF =)
Reference solar cell	Ref. cell ID	KIER-SS-FD #1
	Package type & materials	Crystalline silicon<br>(KG3 color filter window)
	Calibration value (I_{sc} under STC)	67.02 mA
	Calibration due date	2022.06.18

No.	Area (cm^2)	V_{oc} (V)	I_{sc} (mA)	fill factor[1]	efficiency[2] (%)	P_{max}[2] (mW)
1	0.079 033	1.168	1.963	0.816 6	23.69	1.872

* See appendix for detailed data.

* Device ID

1 : KIM #4 [Appendix 1]

* Comments

1. The measurement results in this document were obtained using the shading mask provided by the client. Hence, if the surface material or aperture area of the mask is changed, the measurement results can be different from the values in this document.

1) fill factor = P_{max} / (V_{oc} x I_{sc})
2) efficiency = P_{max} / (Input irradiance x Area) [According to the client request, uncertainty analysis was omitted.]
3) P_{max} = Maximum value of "applied voltage x output current of the test specimen" under the test condition.

KIER-QP-22-02-B(Rev.12)

0844-1386-6403-6267

KIER

Test Results

Report No. : KIER-211221012
Page(4)/(4)Pages

KOLAS — KOREA LABORATORY ACCREDITATION SCHEME — TESTING NO.KT201

[Appendix 1]

Ecole Polytechnique Federale de Lausanne (EPFL)
Perovskite solar cell

Device ID : KIM #4
Date of Test : January 18, 2022
Simulator : WACOM, WXS-155S-L2 (Class-AAA)
Reference solar cell : KIER-SS-FD #1
Test condition : STC [AM1.5G, 100 mW/cm^2, (25.0 ± 1.0) °C]

Device Area : 0.079 033 cm^2 (designated illumination area)
Sample Type : Perovskite solar cell (glass plate based)

한국에너지기술연구원
KOREA INSTITUTE OF ENERGY RESEARCH

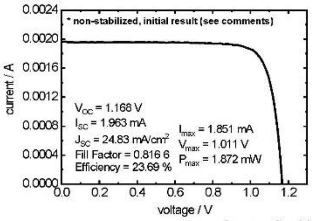

Operator: SangMin Lee

Photovoltaics Research Department, Korea Institute of Energy Research
152, Gajeong-ro, Yuseong-gu, Daejeon, 34129, Korea
Tel : +82-42-860-3182, e-mail : notask@kier.re.kr

\- End -

KIER-QP-22-02-B(Rev.12)

0844-1386-6403-6267

Supplementary Fig. 24.

Certificate of PSCs using the 3C/6H hetero-polytypic perovskite with the LDP passivation. The certified efficiency of 23.69% was confirmed by Korea Institute of Energy Research in Republic of Korea. This certificate is provided with written permission from Korea Institute of Energy Research.

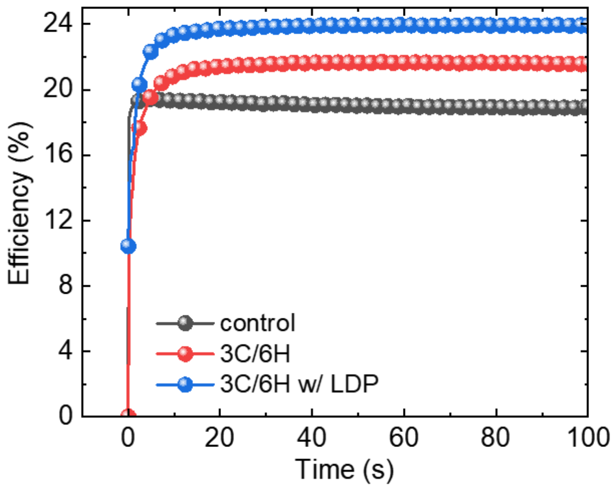

Supplementary Fig. 25.

Steady-state efficiency of using the control perovskite (black), the 3C/6H hetero-polytypic perovskite (red), and the 3C/6H hetero-polytypic perovskite with surface passivation (blue) by tracking maximum power point.

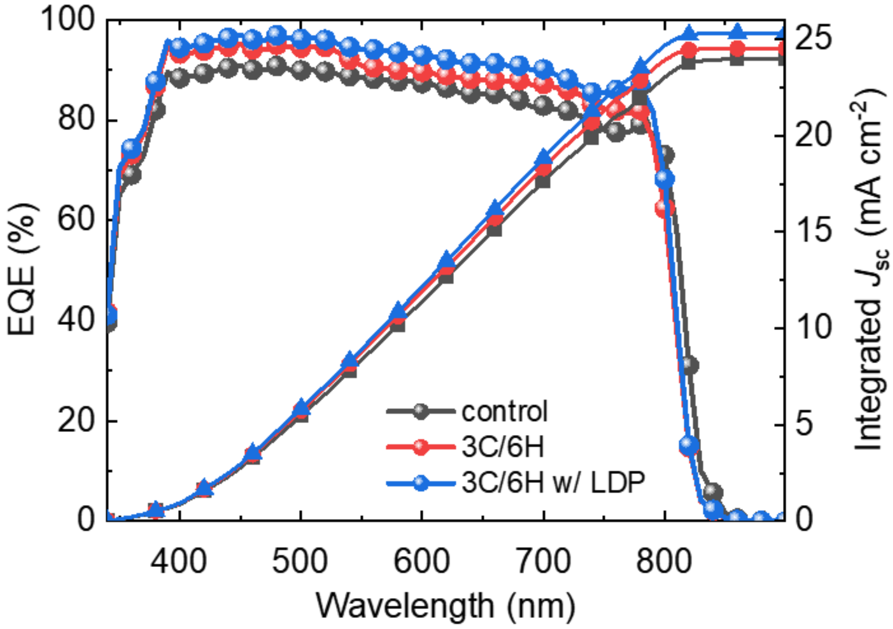

Supplementary Fig. 26.

External quantum efficiency (EQE) spectra of PSCs using the control perovskite (black), the 3C/6H hetero-polytypic perovskite (red), and the 3C/6H hetero-polytypic perovskite with surface passivation (blue). The integrated J_{sc} of 23.97, 24.53, and 25.33 mA cm^{-2} were obtained from the EQE spectra, respectively.

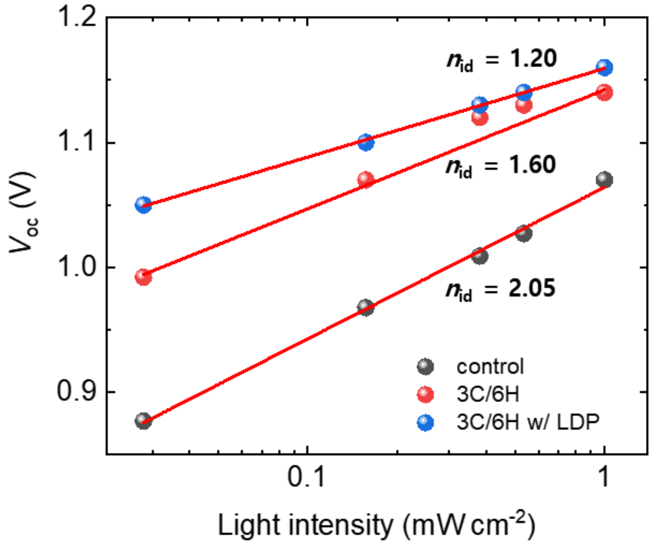

Supplementary Fig. 27.

Based on the light intensity dependent V_{oc}, ideality factors (n_{id}) of PSCs were extracted for the control perovskite (black), the 3C/6H hetero-polytypic perovskite (red), and the 3C/6H hetero-polytypic perovskite with surface passivation (blue). The control device resulted in an n_{id} of 2.05, whereas the 6H polytype decreased n_{id} to 1.60 and the additional LDP passivation further decreased n_{id} to 1.20. As n_{id} = 1 is expected for a film to have only bimolecular radiative recombination and n_{id} = 2 to have monomolecular nonradiative recombination[41], decreasing n_{id} indicates that the use of the 6H-bridged film and the additional surface passivation layer suppresses non-radiative recombination in the films.

mks Newport	ACCREDITED Calibration Cert. # 2893.01	Technology and Application Center **PV Lab** Newport Calibration Cert. # 2657

DUT S/N: 7286555 D1
Newport Calibration #: 2657
Manufacturer: EPFL/ Gwangju Institute of Science /Technology Korea Research Institute of Chemical Technology
Material (single junction): Perovskite
Measurement Date: 26-SEP-2022
Temperature Sensor: TC-K, DUT Temperature: 24.9 ± 0.7 ˚C
Environmental conditions at the time of calibration: Temperature: 24 ± 3 ˚C; Humidity: 30 ± 20 %

The above DUT has been tested using the following methods to meet the ISO 17025 Standard by the PV Lab at Newport Corporation. Quoted uncertainties are expanded using a coverage factor of $k = 2$ and expressed with an approximately 95% level of confidence. Measurement of total irradiance is traceable to the World Radiometric Reference (WRR) and all other measurements and uncertainties are traceable to NIST and the International System of Units (SI). The performance parameters reported in this certificate apply only at the time of the test, and do not imply future performance.

* **Designated area as thin metal aperture.**

† **Reported performance parameters below are average of reverse (open-circuit → short-circuit) and forward (short-circuit → open-circuit) IV sweep results at a sweep rate of 200 mV/s, and *do not represent the device behavior under quasi-steady-state conditions*. At this sweep rate, hysteresis of +/- 5.449% of average PCE was observed.**

Efficiency [%]	20.4†	±	1.5	V_oc [V]	8.93	±	0.14	I_sc [A]	0.0892	±	0.0019
P_max [mW]	583	±	41	V_max [V]	7.11	±	0.33	I_max [A]	0.0820	±	0.0025
FF [%]	73.2	±	3.9	Area [cm²]	28.617*	±	0.061	M	1.011	±	0.015

Methods:
I-V: ASTM E948-16 *Standard Test Method for Electrical Performance of Photovoltaic Cells Using Reference Cells Under Simulated Sunlight*
QE: ASTM E1021-15 *Standard Test Method for Spectral Responsivity Measurements of Photovoltaic Devices*

Standard Reporting Conditions:
Spectrum: AM1.5-G (ASTM G173-03/IEC 60904-3 ed. 2)
1000.0 W/m ² at 25.0˚C

Secondary Reference Cell:
Device S/N: PVM 746
Device Material: GaAs
Window Material: BK7
Certification: National Renewable Energy Laboratory
A2LA accreditation certificate # 2236.01
ISO Tracking #: 2035
Certified short circuit current (I_{sc}) under standard reporting conditions (SRC): 105.87 mA
Calibration due date: 23-Apr-23

Solar Simulator:
Spectrum: Newport Corporation filename *Sol3A_Spectroradiometer_Scan_0229.xls*
Total irradiance: 1000 W/m² based on I_{sc} of the above Secondary Reference Cell

Quantum Efficiency for DUT:
Newport Corporation filename *QE 7286555 D1, Dark.log*
Spectral mismatch correction factor: M = 1.011 ± 0.015

DUT Calibration Procedures:
Newport Corporation document WI1 (EQE).docx
Newport Corporation document Area Measurement WI2 (Area).docx
Newport Corporation document WI3 (IV.Sweep).docx

Cal Cert V1.8	Issue Date: Nov 2, 2022	Page 2 of 2
	Reviewed and Approved by: Paulette Frischknecht (Paulette.Frischknecht@mksinst.com)	
This certificate to be reproduced in part only with written permission from the Newport PV Laboratory		

Supplementary Fig. 28.

Certificate of the perovskite solar module using the 3C/6H hetero-polytypic perovskite with LDP passivation, which was issued by Newport Photovoltaic Testing and Calibration Laboratory in the US. This certificate is provided with written permission from Newport.

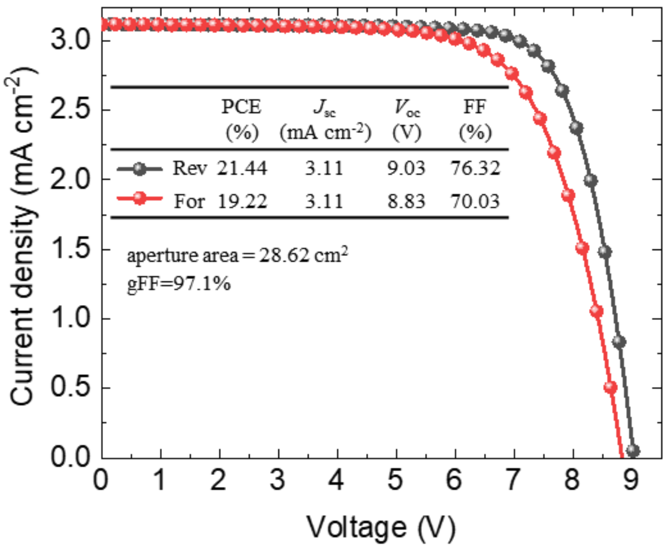

Supplementary Fig. 29.

J-*V* characteristics of the certified device in the reverse scan and forward scan, respectively, resulting in the average PCE of 20.4%.

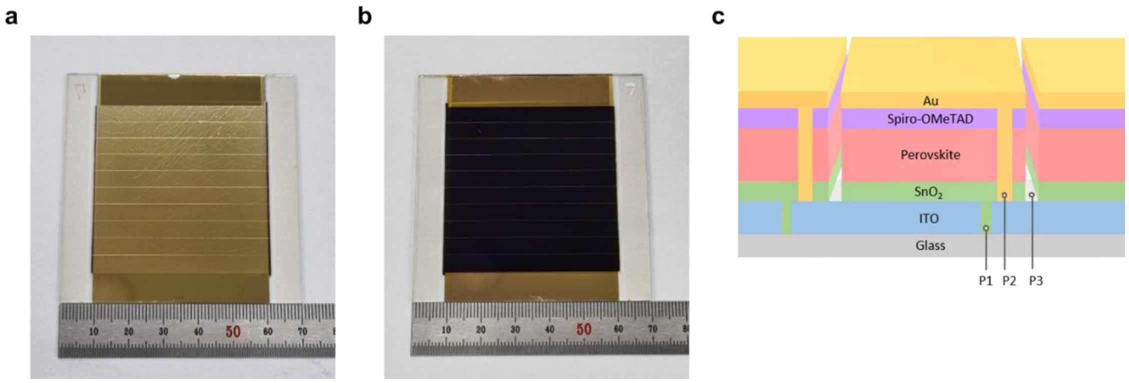

Supplementary Fig. 30.

(**a**) Front and (**b**) back side of a 7 cm × 7 cm of perovskite solar module. (**c**) Schematic illustration of the perovskite solar module realized by P1-P2-P3 interconnection.

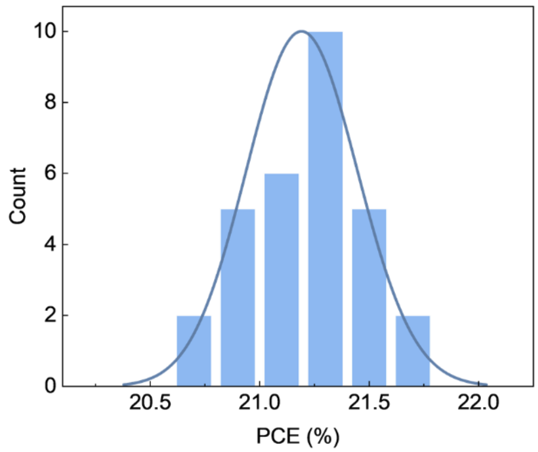

Supplementary Fig. 31.
Histogram of the PCEs for 30 low-temperature processed perovskite solar modules.

Supplementary Table 11.

J-V characteristics of 30 perovskite solar modules fabricated using the shearing coating method.

V_{oc} (V)	J_{sc} (mA/cm^2)	FF	PCE (%)
12.10	2.20	79.67	21.23
12.10	2.20	79.27	21.14
12.10	2.21	78.59	21.02
12.18	2.21	78.40	21.10
12.18	2.22	77.84	21.02
12.18	2.22	78.69	21.30
12.18	2.22	78.61	21.24
12.10	2.22	78.68	21.16
12.10	2.21	77.77	20.82
12.10	2.15	80.33	20.90
12.10	2.22	78.06	20.99
12.18	2.22	79.98	21.62
12.18	2.22	79.74	21.55
12.18	2.21	77.56	20.92
12.10	2.15	80.00	20.78
12.18	2.22	79.67	21.52
12.10	2.21	80.13	21.48
12.10	2.22	79.91	21.42
12.10	2.21	79.57	21.30
12.18	2.23	79.96	21.74
12.18	2.21	78.90	21.20
12.10	2.20	79.58	21.21
12.10	2.21	79.45	21.20
12.10	2.20	79.37	21.14
12.18	2.20	79.37	21.25
12.26	2.25	77.74	21.45
12.10	2.21	80.07	21.37
12.10	2.15	79.75	20.77
12.18	2.19	79.36	21.20
12.18	2.22	77.09	20.82

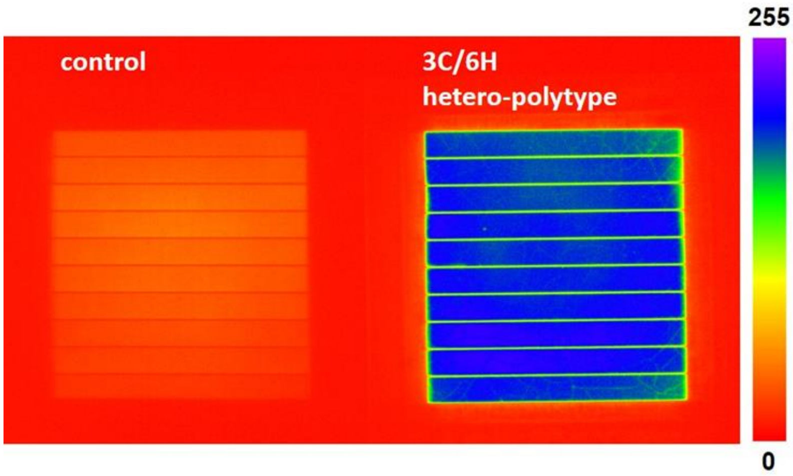

Supplementary Fig. 32.

PL imaging of large-area films composed of the control perovskite and the 3C/6H hetero-polytypic perovskite.

Supplementary Table 12.

Summary of PCEs in our work.

	**High-temp. process spin-coating**		**Low-temp. process shearing-coating**	
Device structure	FTO/c-TiO_2/m-TiO_2 /KCl/perovskite/OAI/Spiro/Au		ITO/Sn(acac)$_2$$Cl_2$/$SnO_2$ /Li_2CO_3/perovskite/OAI/Spiro/Au	
Unit-cell (Lab PCE)	24.13%		-	
Unit-cell (certified PCE, KIER)	23.69%		-	
Module (Lab PCE)	Aperture	Active	Aperture	Active
	21.92%	22.57%	21.74%	22.96%
Module (certified PCE, Newport)	Aperture	Active	-	
	21.44%	22.08%		

* Active PCE of module is calculated based on geometric fill factor.

Supplementary Table 13.

Overview of recently reported perovskite solar modules with an area of ≥ 20cm^2. ap, aperture area; ac, active area; da, designated illumination area; ac-gFF, active area calculated based on gFF. [a]National Photovoltaic Product Quality Supervision & Inspection Center, China; [b]NREL, National Renewable Energy Laboratory, US; [c]National Institute of Metrology, China; [d] Newport, Newport corporation, US; [e]Daegu Technopark Nano Convergence Practical Application Center, Republic of Korea; [f]OMA, OMA company (Newport Korea distributor); [g]Photovoltaic and Wind Power Systems Quality Test Center, IEE, Chinese Academy of Sciences; [h]National Photovoltaic Industry Metrology and Testing Center.

**Year**	**Area (cm^2)**	**gFF (%)**	**Number of subcell**	***J*sc (mA/cm^2)**	***V*oc (V)**	**FF (%)**	**PCE (%)**	**Test center**	**ref**
2018	33[ap]	93.4	17	1.15	18.17	72.1	15.0[ap]	-	42
2019	24.97[da]	94.4	8	2.72	8.78	71.7	17.1[da]	-	43
2020	36.1[ac]	85	10	1.77	11.19	64.91	12.4[ac]	-	44
2020	36[ap]	-	10	1.8	10.4	67.67	12.67[ap]	-	45
2020	25.49[ap]	90.8	7	3.03	7.52	78.59	17.88[ap]	NPQSIC[a]	46
2020	22.4[da]	91	7	2.99	7.64	72.9	16.6[da]<br>18.2[ac]	-	47
2021	35.8[ap]	-	10	2.04	11.7	77.3	18.5[ap]	-	48
2021	36.6[ac]	90.3	7	3.04	7.44	71	16.06[ac]	-	49
2021	42.8[ac]	-	14	1.5	16.05	70.89	17.05[ac]	-	50
2021	29.539[da]	92	8	2.83	8.71	75.41	18.6[da]	NREL[b]	51
2021	31[ap]	94.36	10	2.14	11.79	80.77	20.4[ap]	-	52
	30.98[ap]	-	10	2.30	11.67	65.4	17.53[ap]	Newport[d]	
2021	26[ac]	90.2	9	2.71	10.3	76.4	19.27[da]<br>21.36[ac]	-	53
2022	25[ac]	-	10	2.35	11.81	78.54	21.83[ac]	-	54
	25[ac]	-	10	2.35	11.6	78.51	21.45[ac]	DGTP[e]	
2022	20[ac]	95.6	10	2.29	12.15	77.9	21.66[ac]	OMA[f]	12
2022	34.36[ap]	88	8	3.24	9.368	74.5	19.88[ap]<br>22.59[ac-gFF]	-	55
2022	22.4[ap]	96	7	3.16	8.13	80	20.5[ap]<br>21.4[ac-gFF]	-	56
2022	24.63[ac]	82	9	2.75	10.155	82	22.87[ac]	-	57
	23.9[ac]	-	9	2.74	10.505	79.06	22.72[ac]	IEE[g]	
2022	30.77[ap]	91	8	3.08	8.761	79.1	19.43[ap]<br>21.35[ac-gFF]	-	58
2022	26.02[da]	-	8	25.61	1.127	77.6	22.4[da]	NPVM[h]	59
2024	31[ap]	91	9	2.7	10.05	80	21.8[ap]<br>24.0[ac-gFF]	-	60
	31[ap]	91	9	2.7	10.03	79.7	21.6[ap]	NPVM	
2024	28.62[ap]	97.1	8	3.1	9.04	77.05	21.92[ap]<br>22.57[ac-gFF]	-	This work
	28.62[ap]	97.1	8	3.1	9.03	76.32	21.44[ap]<br>22.08[ac-gFF]	Newport	
	24.5[ap]	94.7	10	2.23	12.18	79.96	21.74[ap]<br>22.96[ac-gFF]	-	

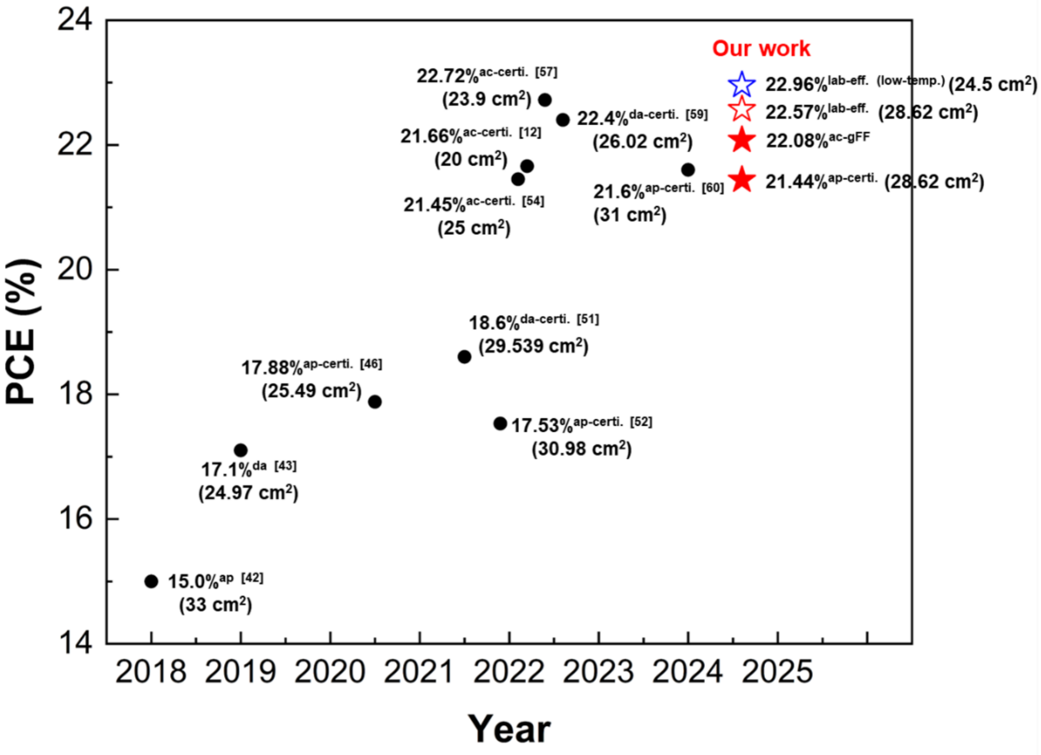

Supplementary Fig. 33.

Evolution of PCEs in perovskite solar modules with an area of ≥ 20cm^2.

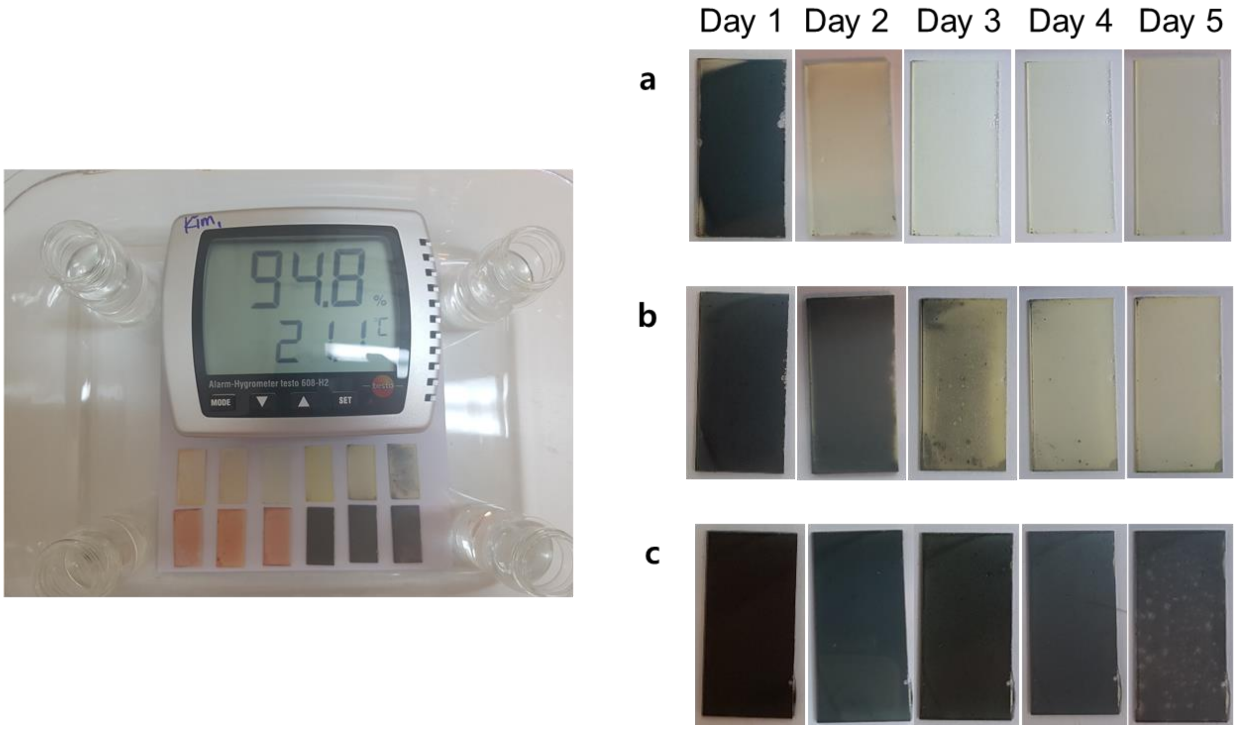

Supplementary Fig. 34.

Moisture stability test of (**a**) the control film, (**b**) the 3C/6H hetero-polytypic perovskite film, and (**c**) the 3C/6H hetero-polytypic perovskite film with surface passivation. Perovskite films were exposed to relative humidity (RH) of 90±5% at room temperature in the dark.

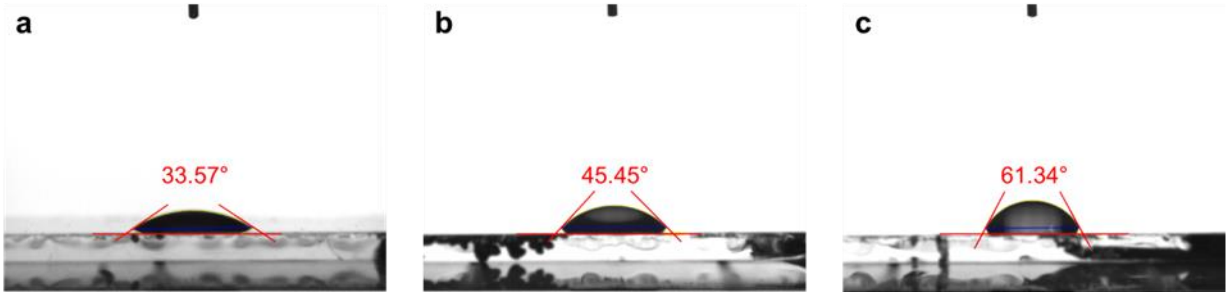

Supplementary Fig. 35.

Water contact angle of the films of (a) control perovskite, (b) 3C/6H hetero-polytypic perovskite, and (c) 3C/6H hetero-polytypic perovskite with LDP passivation.

Supplementary References

1. Bi, D. *et al.* Efficient luminescent solar cells based on tailored mixed-cation perovskites. *Sci. Adv.* **2**, e1501170 (2016).
2. Kim, Y. C. *et al.* Beneficial Effects of PbI2 Incorporated in Organo-Lead Halide Perovskite Solar Cells. *Advanced Energy Materials* **6**, 1502104 (2016).
3. Chen, S. *et al.* Spatial Distribution of Lead Iodide and Local Passivation on Organo-Lead Halide Perovskite. *ACS Appl. Mater. Interfaces* **9**, 6072–6078 (2017).
4. Park, B. *et al.* Understanding how excess lead iodide precursor improves halide perovskite solar cell performance. *Nat Commun* **9**, 3301 (2018).
5. Zhang, D. *et al.* Stable α-FAPbI3 in Inverted Perovskite Solar Cells with Efficiency Exceeding 22% via a Self-Passivation Strategy. *Advanced Functional Materials* **32**, 2200174 (2022).
6. Zhong, H. *et al.* Suppressing the crystallographic disorders induced by excess PbI2 to achieve trade-off between efficiency and stability for PbI2-rich perovskite solar cells. *Nano Energy* **105**, 108014 (2023).
7. Zhang, H. *et al.* Excess PbI2 Management via Multimode Supramolecular Complex Engineering Enables High-Performance Perovskite Solar Cells. *Advanced Energy Materials* **12**, 2201663 (2022).
8. Tan, S. *et al.* Shallow Iodine Defects Accelerate the Degradation of α-Phase Formamidinium Perovskite. *Joule* **4**, 2426–2442 (2020).
9. Jeong, J. *et al.* Pseudo-halide anion engineering for α-FAPbI3 perovskite solar cells. *Nature* **592**, 381–385 (2021).
10. Yang, G., Zhang, H., Li, G. & Fang, G. Stabilizer-assisted growth of formamdinium-based perovskites for highly efficient and stable planar solar cells with over 22% efficiency. *Nano Energy* **63**, 103835 (2019).

11. Kim, M. *et al.* Methylammonium Chloride Induces Intermediate Phase Stabilization for Efficient Perovskite Solar Cells. *Joule* **3**, 2179–2192 (2019).

12. Kim, M. *et al.* Conformal quantum dot–SnO_2 layers as electron transporters for efficient perovskite solar cells. *Science* **375**, 302–306 (2022).

13. Zhao, Y. *et al.* Double-Side-Passivated Perovskite Solar Cells with Ultra-low Potential Loss. *Solar RRL* **3**, 1800296 (2019).

14. von Weimarn, P. P. The Precipitation Laws. *Chem. Rev.* **2**, 217–242 (1925).

15. Liu, D., Zhou, W., Tang, H., Fu, P. & Ning, Z. Supersaturation controlled growth of MAFAPbI3 perovskite film for high efficiency solar cells. *Sci. China Chem.* **61**, 1278–1284 (2018).

16. Yang, W. S. *et al.* High-performance photovoltaic perovskite layers fabricated through intramolecular exchange. *Science* **348**, 1234–1237 (2015).

17. Kaiser, W. *et al.* Halide-driven formation of lead halide perovskites: insight from ab initio molecular dynamics simulations. *Mater. Adv.* **2**, 3915–3926 (2021).

18. Ahlawat, P. *et al.* A combined molecular dynamics and experimental study of two-step process enabling low-temperature formation of phase-pure α-FAPbI3. *Science Advances* **7**, eabe3326 (2021).

19. Lee, J.-W. *et al.* Formamidinium and Cesium Hybridization for Photo- and Moisture-Stable Perovskite Solar Cell. *Advanced Energy Materials* **5**, 1501310 (2015).

20. Binek, A., Hanusch, F. C., Docampo, P. & Bein, T. Stabilization of the Trigonal High-Temperature Phase of Formamidinium Lead Iodide. *J. Phys. Chem. Lett.* **6**, 1249–1253 (2015).

21. Wu, J. *et al.* Regioselective Multisite Atomic-Chlorine Passivation Enables Efficient and Stable Perovskite Solar Cells. *J. Am. Chem. Soc.* **145**, 5872–5879 (2023).

22. Hang, P. *et al.* An Interlayer with Strong Pb-Cl Bond Delivers Ultraviolet-Filter-Free, Efficient, and Photostable Perovskite Solar Cells. *iScience* **21**, 217–227 (2019).

23. Momma, K. & Izumi, F. VESTA 3 for three-dimensional visualization of crystal, volumetric and morphology data. *J Appl Cryst* **44**, 1272–1276 (2011).

24. Stoumpos, C. C., Malliakas, C. D. & Kanatzidis, M. G. Semiconducting Tin and Lead Iodide Perovskites with Organic Cations: Phase Transitions, High Mobilities, and Near-Infrared Photoluminescent Properties. *Inorganic Chemistry* **52**, 9019–9038 (2013).

25. Jeon, N. J. *et al.* Compositional engineering of perovskite materials for high-performance solar cells. *Nature* **517**, 476–480 (2015).

26. Chen, T. *et al.* Entropy-driven structural transition and kinetic trapping in formamidinium lead iodide perovskite. *Science Advances* **2**, e1601650 (2016).

27. Lee, J.-W. *et al.* Solid-phase hetero epitaxial growth of α-phase formamidinium perovskite. *Nat Commun* **11**, 5514 (2020).

28. Perdew, J. P., Burke, K. & Ernzerhof, M. Generalized Gradient Approximation Made Simple. *Phys. Rev. Lett.* **77**, 3865–3868 (1996).

29. VandeVondele, J. & Hutter, J. Gaussian basis sets for accurate calculations on molecular systems in gas and condensed phases. *J. Chem. Phys.* **127**, 114105 (2007).

30. Goedecker, S., Teter, M. & Hutter, J. Separable dual-space Gaussian pseudopotentials. *Phys. Rev. B* **54**, 1703–1710 (1996).

31. Perdew, J. P., Ernzerhof, M. & Burke, K. Rationale for mixing exact exchange with density functional approximations. *J. Chem. Phys.* **105**, 9982–9985 (1996).

32. Adamo, C. & Barone, V. Toward reliable density functional methods without adjustable parameters: The PBE0 model. *J. Chem. Phys.* **110**, 6158–6170 (1999).

33. Guidon, M., Hutter, J. & VandeVondele, J. Auxiliary Density Matrix Methods for Hartree−Fock Exchange Calculations. *J. Chem. Theory Comput.* **6**, 2348–2364 (2010).

34. Syzgantseva, O. A., Saliba, M., Grätzel, M. & Rothlisberger, U. Stabilization of the Perovskite Phase of Formamidinium Lead Triiodide by Methylammonium, Cs, and/or Rb Doping. *J. Phys. Chem. Lett.* **8**, 1191–1196 (2017).

35. Kühne, T. D. *et al.* CP2K: An electronic structure and molecular dynamics software package - Quickstep: Efficient and accurate electronic structure calculations. *J. Chem. Phys.* **152**, 194103 (2020).

36. Syzgantseva, M. A., Stepanov, N. F. & Syzgantseva, O. A. Carrier Lifetimes and Recombination Pathways in Metal–Organic Frameworks. *J. Phys. Chem. Lett.* **10**, 5041–5046 (2019).

37. Wright, A. D. *et al.* Electron–phonon coupling in hybrid lead halide perovskites. *Nat Commun* **7**, 11755 (2016).

38. Han, Q. *et al.* Single Crystal Formamidinium Lead Iodide ($FAPbI_3$): Insight into the Structural, Optical, and Electrical Properties. *Adv. Mater.* **28**, 2253–2258 (2016).

39. Saidaminov, M. I. *et al.* High-quality bulk hybrid perovskite single crystals within minutes by inverse temperature crystallization. *Nat Commun* **6**, 7586 (2015).

40. Dong, Q. *et al.* Electron-hole diffusion lengths > 175 μm in solution-grown $CH_3NH_3PbI_3$ single crystals. *Science* **347**, 967–970 (2015).

41. Caprioglio, P. *et al.* On the Origin of the Ideality Factor in Perovskite Solar Cells. *Adv. Energy Mater.* **10**, 2000502 (2020).

42. Deng, Y. *et al.* Surfactant-controlled ink drying enables high-speed deposition of perovskite films for efficient photovoltaic modules. *Nat Energy* **3**, 560–566 (2018).

43. Jung, E. H. *et al.* Efficient, stable and scalable perovskite solar cells using poly(3-hexylthiophene). *Nature* **567**, 511–515 (2019).

44. Ru, P. *et al.* High Electron Affinity Enables Fast Hole Extraction for Efficient Flexible Inverted Perovskite Solar Cells. *Advanced Energy Materials* **10**, 1903487 (2020).

45. Li, E. *et al.* Synergistic Coassembly of Highly Wettable and Uniform Hole-Extraction Monolayers for Scaling-up Perovskite Solar Cells. *Advanced Functional Materials* **30**, 1909509 (2020).

46. Ren, A. *et al.* Efficient Perovskite Solar Modules with Minimized Nonradiative Recombination and Local Carrier Transport Losses. *Joule* **4**, 1263–1277 (2020).

47. Liu, Z. *et al.* A holistic approach to interface stabilization for efficient perovskite solar modules with over 2,000-hour operational stability. *Nat Energy* **5**, 596–604 (2020).

48. Chen, S., Xiao, X., Gu, H. & Huang, J. Iodine reduction for reproducible and high-performance perovskite solar cells and modules. *Sci. Adv.* **7**, eabe8130 (2021).

49. Huang, H.-H. *et al.* A simple one-step method with wide processing window for high-quality perovskite mini-module fabrication. *Joule* **5**, 958–974 (2021).

50. Yaghoobi Nia, N. *et al.* Beyond 17% stable perovskite solar module via polaron arrangement of tuned polymeric hole transport layer. *Nano Energy* **82**, 105685 (2021).

51. Deng, Y. *et al.* Defect compensation in formamidinium–caesium perovskites for highly efficient solar mini-modules with improved photostability. *Nat Energy* **6**, 633–641 (2021).

52. Yoo, J. W. *et al.* Efficient perovskite solar mini-modules fabricated via bar-coating using 2-methoxyethanol-based formamidinium lead tri-iodide precursor solution. *Joule* **5**, 2420–2436 (2021).

53. Liu, C. *et al.* Tuning structural isomers of phenylenediammonium to afford efficient and stable perovskite solar cells and modules. *Nat Commun* **12**, 6394 (2021).

54. Jeong, M. *et al.* Large-area perovskite solar cells employing spiro-Naph hole transport material. *Nat. Photon.* **16**, 119–125 (2022).

55. Liu, X. *et al.* Area-Scalable Zn2SnO4 Electron Transport Layer for Highly Efficient and Stable Perovskite Solar Modules. *ACS Appl. Mater. Interfaces* **14**, 23297–23306 (2022).

56. Bu, T. *et al.* Modulating crystal growth of formamidinium–caesium perovskites for over 200 cm2 photovoltaic sub-modules. *Nat Energy* **7**, 528–536 (2022).

57. Ding, Y. *et al.* Single-crystalline TiO2 nanoparticles for stable and efficient perovskite modules. *Nat. Nanotechnol.* **17**, 598–605 (2022).

58. Xia, J. *et al.* Asymmetrically Substituted 10H,10′H-9,9′-Spirobi[acridine] Derivatives as Hole-Transporting Materials for Perovskite Solar Cells. *Angewandte Chemie International Edition* **61**, e202212891 (2022).

59. Ding, B., Yi, Z. & Ding, Y. Development of efficient and stable perovskite solar cells and modules. Fifth International Conference on Materials & Environmental Science (ICMES-2022), June 09–12, 2022, Saïdia, Morocco.

60. Yang, Y. *et al.* A thermotropic liquid crystal enables efficient and stable perovskite solar modules. *Nat Energy* 1–8 (2024) doi:10.1038/s41560-023-01444-z.
