# Supplementary material for: Shallow-level defect passivation by 6H perovskite polytype for highly efficient and stable perovskite solar cells
Source: Nat Commun. 2024 Jul 4;15:5632. doi: 10.1038/s41467-024-50016-6 (PMC11224362; doi:10.1038/s41467-024-50016-6)
Supplement: Supplementary file 3 — Description of Additional Supplementary Files [file 41467_2024_50016_MOESM3_ESM.pdf]

File name: Supplementary Movie 1

Description: Molecular dynamics simulations of the 3C/6H interface.
