# Supplementary material for: Shallow-level defect passivation by 6H perovskite polytype for highly efficient and stable perovskite solar cells
Source: Nat Commun. 2024 Jul 4;15:5632. doi: 10.1038/s41467-024-50016-6 (PMC11224362; doi:10.1038/s41467-024-50016-6)
Supplement: Supplementary file 5 — Reporting Summary [file 41467_2024_50016_MOESM5_ESM.pdf]

nature portfolio

Corresponding author(s): Hobeom Kim, Nam Joong Jeon, Paul J. Dyson, and Mohammad K. Nazeeruddin

Solar Cells Reporting Summary

Nature Portfolio wishes to improve the reproducibility of the work that we publish. This form is intended for publication with all accepted papers reporting the characterization of photovoltaic devices and provides structure for consistency and transparency in reporting. Some list items might not apply to an individual manuscript, but all fields must be completed for clarity.

For further information on Nature Research policies, including our data availability policy, see Authors & Referees.

Experimental design

Please check the following details are reported in the manuscript, and provide a brief description or explanation where applicable.

1. Dimensions

Area of the tested solar cells

☒ Yes
☐ No

The active area of small-sized devices and modules were 0.0804 cm2, 27.79cm2 and 23.2 cm2, respectively.

Explain why this information is not reported/not relevant.

Method used to determine the device area

☒ Yes
☐ No

Characterization part in Method.

Explain why this information is not reported/not relevant.

2. Current-voltage characterization

Current density-voltage (J-V) plots in both forward and backward direction

☒ Yes
☐ No

Supplementary Fig. 23

Voltage scan conditions

☒ Yes
☐ No

Characterization part in Method.

Explain why this information is not reported/not relevant.

Test environment

☒ Yes
☐ No

Characterization part in Method.

Explain why this information is not reported/not relevant.

Protocol for preconditioning of the device before its characterization

☐ Yes
☒ No

Provide a description of the protocol.

No preconditioning of the device was applied before its characterization.

Stability of the J-V characteristic

☒ Yes
☐ No

Fig. 5e and Supplementary Fig. 25

Explain why this information is not reported/not relevant.

3. Hysteresis or any other unusual behaviour

Description of the unusual behaviour observed during the characterization

☐ Yes
☒ No

Provide a description of hysteresis or any other unusual behaviour observed during the characterization.

No unusual hysteresis behavior was observed.

Related experimental data

☐ Yes
☒ No

Provide a description of the related experimental data.

No unusual hysteresis behavior was observed.

4. Efficiency

External quantum efficiency (EQE) or incident photons to current efficiency (IPCE)

☒ Yes
☐ No

Supplementary Fig. 26

Explain why this information is not reported/not relevant.

A comparison between the integrated response under the standard reference spectrum and the response measure under the simulator

☒ Yes
☐ No

Supplementary Fig. 26

Explain why this information is not reported/not relevant.

For tandem solar cells, the bias illumination and bias voltage used for each subcell

☐ Yes
☒ No

Provide a description of the measurement conditions.

No tandem solar cell is reported in this work.

5. Calibration

Light source and reference cell or sensor used for the characterization

☒ Yes
☐ No

Characterization part in Method.

Explain why this information is not reported/not relevant.

Confirmation that the reference cell was calibrated and certified

☒ Yes
☐ No

The Si reference cell (KG3) was calibrated by Newport. Characterization part in Method.

Explain why this information is not reported/not relevant.

Calculation of spectral mismatch between the reference cell and the devices under test

☐ Yes
☒ No

Provide a value of the spectral mismatch and/or a description of how it has been taken into account in the measurements.

No spectral mismatch calculation was performed.

6. Mask/aperture

Size of the mask/aperture used during testing

☒ Yes
☐ No

Characterization part in Method.

Explain why this information is not reported/not relevant.

Variation of the measured short-circuit current density with the mask/aperture area

☒ Yes
☐ No

Some modules were measured with different apertures, including 24.5 and 28.62 cm2.

Explain why this information is not reported/not relevant.

7. Performance certification

Identity of the independent certification laboratory that confirmed the photovoltaic performance

☒ Yes
☐ No

Supplementary Fig. 24 and Supplementary Fig. 28

Explain why this information is not reported/not relevant.

A copy of any certificate(s)

☒ Yes
☐ No

Supplementary Fig. 24 and Supplementary Fig. 28

Explain why this information is not reported/not relevant.

8. Statistics

Number of solar cells tested

☒ Yes
☐ No

20 cells of each experimental condition (Fig. 5a and Supplementary Table 10), and 30 low-temperature processed modules (Supplementary Fig. 30 and Supplementary Table 11).

Explain why this information is not reported/not relevant.

Statistical analysis of the device performance

☒ Yes
☐ No

Fig. 5a, Supplementary Table 10, Supplementary Fig. 30 and Supplementary Table 11

Explain why this information is not reported/not relevant.

9. Long-term stability analysis

Type of analysis, bias conditions and environmental conditions

☒ Yes
☐ No

Fig. 5e and Characterization part in Method.

Explain why this information is not reported/not relevant.
